# Preventing Loss of Independence through Exercise (PLIÉ): A Pilot Clinical Trial in Older Adults with Dementia

**DOI:** 10.1371/journal.pone.0113367

**Published:** 2015-02-11

**Authors:** Deborah E. Barnes, Wolf Mehling, Eveline Wu, Matthew Beristianos, Kristine Yaffe, Karyn Skultety, Margaret A. Chesney

**Affiliations:** 1 Department of Psychiatry, University of California San Francisco, San Francisco, California, United States of America; 2 Department of Epidemiology & Biostatistics, University of California San Francisco, San Francisco, California, United States of America; 3 San Francisco Veterans Affairs Medical Center, San Francisco, California, United States of America; 4 Department of Family and Community Medicine, University of California San Francisco, San Francisco, California, United States of America; 5 Osher Center for Integrative Medicine, University of California San Francisco, San Francisco, California, United States of America; 6 California Institute of Integral Studies, San Francisco, California, United States of America; 7 Northern California Institute for Research and Education, San Francisco, California, United States of America; 8 California School of Professional Psychology, San Francisco, California, United States of America; 9 Department of Neurology, University of California San Francisco, San Francisco, California, United States of America; 10 Institute on Aging, San Francisco, California, United States of America; 11 Department of Medicine, University of California San Francisco, San Francisco, California, United States of America

## Abstract

**Background:**

Current dementia medications have small effect sizes, many adverse effects and do not change the disease course. Therefore, it is critically important to study alternative treatment strategies. The goal of this study was to pilot-test a novel, integrative group exercise program for individuals with mild-to-moderate dementia called Preventing Loss of Independence through Exercise (PLIÉ), which focuses on training procedural memory for basic functional movements (e.g., sit-to-stand) while increasing mindful body awareness and facilitating social connection.

**Methods:**

We performed a 36-week cross-over pilot clinical trial to compare PLIÉ with usual care (UC) at an adult day program for individuals with dementia in San Francisco, CA. Assessments of physical performance, cognitive function, physical function, dementia-related behaviors, quality of life and caregiver burden were performed by blinded assessors at baseline, 18 weeks (cross-over) and 36 weeks. Our primary outcomes were effect sizes based on between-group comparisons of change from baseline to 18 weeks; secondary outcomes were within-group comparisons of change before and after cross-over.

**Results:**

Twelve individuals enrolled (7 PLIÉ, 5 UC) and 2 withdrew (1 PLIÉ, 18 weeks; 1 UC, 36 weeks). Participants were 82% women (mean age, 84 ± 4 years); caregivers were 82% daughters (mean age, 56 ± 13 years). Effect sizes were not statistically significant but suggested potentially clinically meaningful (≥0.25 SDs) improvement with PLIÉ versus UC for physical performance (Cohen’s D: 0.34 SDs), cognitive function (0.76 SDs) and quality of life (0.83 SDs) as well as for caregiver measures of participant’s quality of life (0.33 SDs) and caregiver burden (0.49 SDs). Results were similar when within-group comparisons were made before and after cross-over.

**Conclusions:**

PLIÉ is a novel, integrative exercise program that shows promise for improving physical function, cognitive function, quality of life and caregiver burden in individuals with mild-to-moderate dementia. Larger randomized, controlled trials are warranted.

**Trial Registration:**

ClinicalTrials.gov NCT01371214

## Introduction

The prevalence of Alzheimer’s disease and other dementias is expected to rise dramatically over the next 40 years at substantial cost to society. There are currently 5.4 million people in the United States[1] and 36 million people worldwide[2] who are living with dementia, which represents one in nine individuals age 65 years or older and one in three age 85 or older.[1] By 2050, prevalence will be 11 to 16 million in the U.S. [1] and 115 million worldwide.[2] Furthermore, the societal costs of caring for individuals with dementia were estimated to be more than $600 billion worldwide in 2010, which is roughly 1% of the global gross domestic product and exceeds the annual budgets of many individual countries.[2] In the U.S. alone, the costs of caring for individuals with dementia were estimated to be $159-$215 billion in 2010, with a projected increase to $379-$511 billion in 2040.[3]

Currently available dementia medications are associated with small improvements in cognitive and physical function (standardized effect sizes, 0.11–0.33) but have many adverse effects and do not stop or slow the disease course.[4–6] In addition, several new medications that initially appeared promising have recently failed in Phase III clinical trials.[7–12] Therefore, it is critically important to study alternative approaches that allow individuals with dementia to maintain physical function, cognitive function and quality of life to the greatest degree possible throughout the disease process.

A growing body of evidence suggests that ‘conventional’ exercise programs such as walking, resistance training and seated exercises that focus on improving aerobic endurance, strength, balance and flexibility have beneficial effects on physical function in individuals with cognitive impairment and dementia.[13–19] A recent meta-analysis identified 16 randomized, controlled trials of conventional exercise interventions in 937 individuals with dementia, finding evidence that exercise improves the ability to perform basic activities of daily living such as eating, dressing, bathing, using the toilet and transferring from bed to chair.[20] However, the effects of conventional exercise on other important outcomes such as cognitive function, mood, behaviors and quality of life were less consistent.

A handful of recent studies suggest that ‘complementary/alternative’ forms of exercise such as tai chi, yoga and dance may be effective for improving these other outcomes. For example, studies have found that tai chi and yoga are associated with improvements in cognitive function and quality of life, as well as physical function, in older adults with and without cognitive impairment.[21–24] In addition, dance-based exercise programs are associated with reductions in problematic behaviors and greater enjoyment in individuals with dementia.[25] Taken together, these prior studies suggest that different types of exercise may offer different benefits, and that a program that combines different approaches may result in greater improvements across multiple domains. In addition, it may be important to incorporate recent evidence from physical and occupational therapy studies, which suggest that a personalized, goal-oriented approach can lead to better outcomes in other settings.[26–28]

Finally, recent discoveries in neuroscience and experimental psychology have found that, although explicit memory (conscious learning of new information) is impaired in individuals with dementia, implicit memory (learning that occurs without conscious awareness) is relatively preserved.[29,30] This is particularly true of implicit memory that involves perceptual or motor learning rather than conceptual learning,[31] including procedural memory (learning to perform procedures).

We hypothesized that an exercise program that focused on training procedural memory to build the strength and capacity to perform the movements that are most needed for daily function (e.g., transitioning safely from sitting to standing) would help individuals with dementia to maintain functional independence. In addition, we hypothesized that the benefits of the program would be enhanced by combining or integrating ‘best elements’ from both conventional and complementary/alternative exercise approaches, particularly through greater in-the-moment body awareness and social connection. We named this integrative exercise program Preventing Loss of Independence through Exercise (PLIÉ). The Guiding Principles of PLIÉ are summarized in Table 1, and the Core Exercise Sequence is included in S1 Appendix.

**Table 1:** Preventing Loss of Independence through Exercise (PLIÉ) Guiding Principles.

Guiding Principles	Rationale	Exercise Approaches Integrated*
1. Repetition with variation	The same basic sequence of events is repeated in each class, providing a structure that becomes familiar over time and is designed to promote procedural learning. Specific movements are varied to maintain engagement based on moment-to-moment participant responses and to include variations introduced by participants.	Physical therapy, occupational therapy, yoga, tai chi, dance movement therapy
2. Progressive, functional movements	Specific movement sequences are selected to be related to important daily functional activities such as standing safely from a seated position. Simpler movements build slowly toward more complexity.	Physical therapy, occupational therapy, Feldenkrais, Rosen
3. Slow pace and step-by-step instructions.	Movements are performed slowly to enable participants to fully process instructions. Step-by-step instruction and modeling are utilized to minimize the cognitive demands and promote procedural learning.	Occupational therapy, yoga, tai chi, Feldenkrais, Rosen, dance movement therapy
4. Participant-centered goal orientation.	A goals assessment is performed before beginning the program. Participants are motivated by relating movements to personal interests and goals.	Physical therapy, occupational therapy
5. Body awareness, mindfulness, and breathing	Periods of rest are included between movements. Participants are encouraged to breathe deeply; notice how their bodies feel aided by sensory stimulation such as tapping, touching and naming body parts; and share their observations with the group.	Yoga, tai chi, Feldenkrais, Rosen
6. Social interaction	Participants sit in a circle, and many movements involve reaching across the circle to touch hands or elbows, or standing in a circle holding hands and moving together to facilitate social connection.	Dance movement therapy, occupational therapy, Rosen
7. Positive emotions	The program promotes positive emotions by creating a warm, loving, non-judgmental, non-coercive environment in which participants are encouraged to move in ways that feel good to them. Brief musical selections are used to enhance positive emotions.	Occupational therapy, yoga, tai chi, Feldenkrais, Rosen method, dance movement therapy

*Physical therapy is a health care profession that focuses on maintaining, restoring and improving movement, activity and health to promote optimal function and quality of life.[53] This is accomplished by examining, evaluating and diagnosing clients and working with them to identify their specific goals and develop an action plan that includes physical exercises such as stretching, strengthening and coordination activities to improve function in daily activities. Physical therapy has a participant-centered goal orientation (Guiding Principal [GP] 4) and exercises are repeated with variations (GP 1) and often involve progressive functional movements (GP 2). Occupational therapy is a health care profession that helps people to participate in the things they want and need to do through the therapeutic use of everyday activities (occupations).[54] It involves performing an individualized evaluation to determine a person’s goals related to functional activities, developing a customized intervention that may include adaptation of the environment as well as specific activities to improve the person’s ability to perform daily activities and reach the goals, and an outcomes evaluation to ensure that the goals are being met and to make changes to the intervention plan as needed, recognizing the functional and social/emotional needs of clients. Occupational therapy has a participant-centered goal orientation (GP 4) and may involve physical exercises that repeat with variations (GP 1) or target progressive functional movements (GP 2); in addition, occupational therapy interventions in people with dementia often utilize a slow pace and step-by-step instruction (GP 3) and emphasize social interaction (GP 6) and positive emotions (GP 7). Yoga is a movement practice from India that seeks to join the mind, body and spirit in a harmonious experience.[55] Yoga primarily includes physical postures, conscious breathing techniques, and meditation practice and sometimes incorporates visualization and the use of sounds or chanting. While hatha yoga is the form of yoga first popularized in the west, there are many forms of yoga, and our study integrated a form of yoga called Healing Yoga[56] that emphasizes nonjudgmental instruction, comfort while moving, and attention to breathing and body sensations. Yoga typically involves repetition of movements with variation (GP 1); a slow pace and step-by-step instruction (GP 3); a focus on body awareness, mindfulness and breathing (GP 6); and promotion of positive emotions (GP 7). Tai chi is a mind-body health practice that originated in China as an internal martial art.[57] It involves performing slow, fluid movement sequences following established forms that are learned over time. Sometimes called ‘moving meditation,’ tai chi practice emphasizes staying aligned, grounded and balanced while moving, with attention to mental and physical relaxation, promoted by deep, diaphragmatic breathing.[58] Tai chi involves repetition of movements with variation (GP 1); a slow pace and step-by-step instruction (GP 3); training of body awareness, mindfulness and breathing (GP 5), and a focus on positive emotions (GP 7). The Feldenkrais Method is a form of somatic (of the body) education that seeks to improve movement, function, range of motion, flexibility and coordination.[59] It is designed to provide an opportunity for neuromuscular re-education through sensory-motor awareness through hundreds of movement sequences called ‘Awareness Through Movement’ that progress in complexity, using variations in positions, attention to body sensation, gentle movement and frequent rests as strategies to change habitual ways of moving, sensing, thinking and feeling.[60] Feldenkrais involves performing basic functional movements that gradually increase in complexity (GP 2); movements are typically taught in a slow, step-by-step manner (GP 3) and are designed to enhance body awareness (GP 5) and promote positive emotions (GP 7). Rosen Method movement classes are set to music and involve slow, easy movements that are designed to improve alignment and flexibility, increase range of motion and ease of breathing, and deepen awareness of the body.[61] The group format of movement classes utilizes social interaction to facilitate a nonjudgmental, relaxed learning environment. It involves learning progressive functional movements (GP 2) in a slow, step-by-step manner (GP 3) with a focus on body awareness, mindfulness and breathing (GP 5), social interaction (GP 6) and positive emotions (GP 7). Dance Movement Therapy is defined as the psychotherapeutic use of movement to promote emotional, social, cognitive and physical integration of the individual.[62] Dance movement therapy in groups with seniors are often in a circle seated formation, usually have a beginning greeting and closing ritual, and involve nonjudgmental explorations combined with verbal processing to facilitate emotional growth and social relatedness.[63] Dance movement therapy includes repetition of dance movement sequences with variations (GP 1), step-by-step instructions (GP 3), and a focus on social interactions (GP 6) and positive emotions (GP 7).

The goal of the current study was to pilot-test the PLIÉ program in order to estimate effect sizes for a larger study by comparing PLIÉ with usual care in 12 individuals who were attending an adult day program in San Francisco, CA. Our pilot-study results suggest that PLIÉ is associated with clinically meaningful improvements (effect sizes ≥ 0.25 standard deviations) in physical function, cognitive function, quality of life and caregiver burden, and that larger randomized, controlled trials are warranted.

## Methods

### Ethics Statement

This trial was approved by the Human Research Protection Program at the University of California, San Francisco (CHR# 10–04080) and is registered at ClinicalTrials.gov (NCT01371214; http://clinicaltrials.gov/ct2/show/record/NCT01371214). The originally approved protocol for this trial and supporting CONSORT checklist are available as supporting information; see S1 Protocol and S1 Checklist. The following changes were approved during the enrollment period: 1) We had originally planned to randomize study participants but were unable to due to small numbers of eligible participants on given days; instead, the PI assigned participants based on their days of attendance and to balance genders between the groups. 2) We relaxed the original inclusion/exclusion criteria to be as inclusive as possible (age > = 55 years, attending center at least 2 days/week, recommended by center staff, English language fluency, caregiver consent and participant assent). 3) Several items from the Senior Fitness Test were added as physical performance measures. 4) The Modified Mini-Mental State Exam[32] was used rather than the Mini-Mental State Exam.[33] 5) Questions related to urinary incontinence were added. 6) The Short Form-36 was dropped for participants, and the Short Form-12 was used for caregivers. 7) Optional monthly home visits were added. 8) Procedures to ensure privacy of data taken offsite were added. After the intervention period had begun, the following additional changes to the study protocol were made: 1) Video recording of a subset of classes was added for the second group. 2) Qualitative data analysis procedures were added. 3) Post-intervention procedures were added.

Informed consent was obtained with the participant and their legally authorized representative together in one meeting. The consent form was reviewed, and participants were asked a series of yes/no questions about the study to assess their capacity to consent. Those who demonstrated capacity to consent signed the consent form for themselves; those who did not demonstrate capacity to consent were asked to assent to the study, and their legally authorized representative signed the consent form on their behalf. Participants who did not assent to study procedures were not eligible to participate. Caregivers signed a separate consent form related to their involvement in the study and could be family members or paid caregivers.

### Overview of Study Design

**Fig 1:**
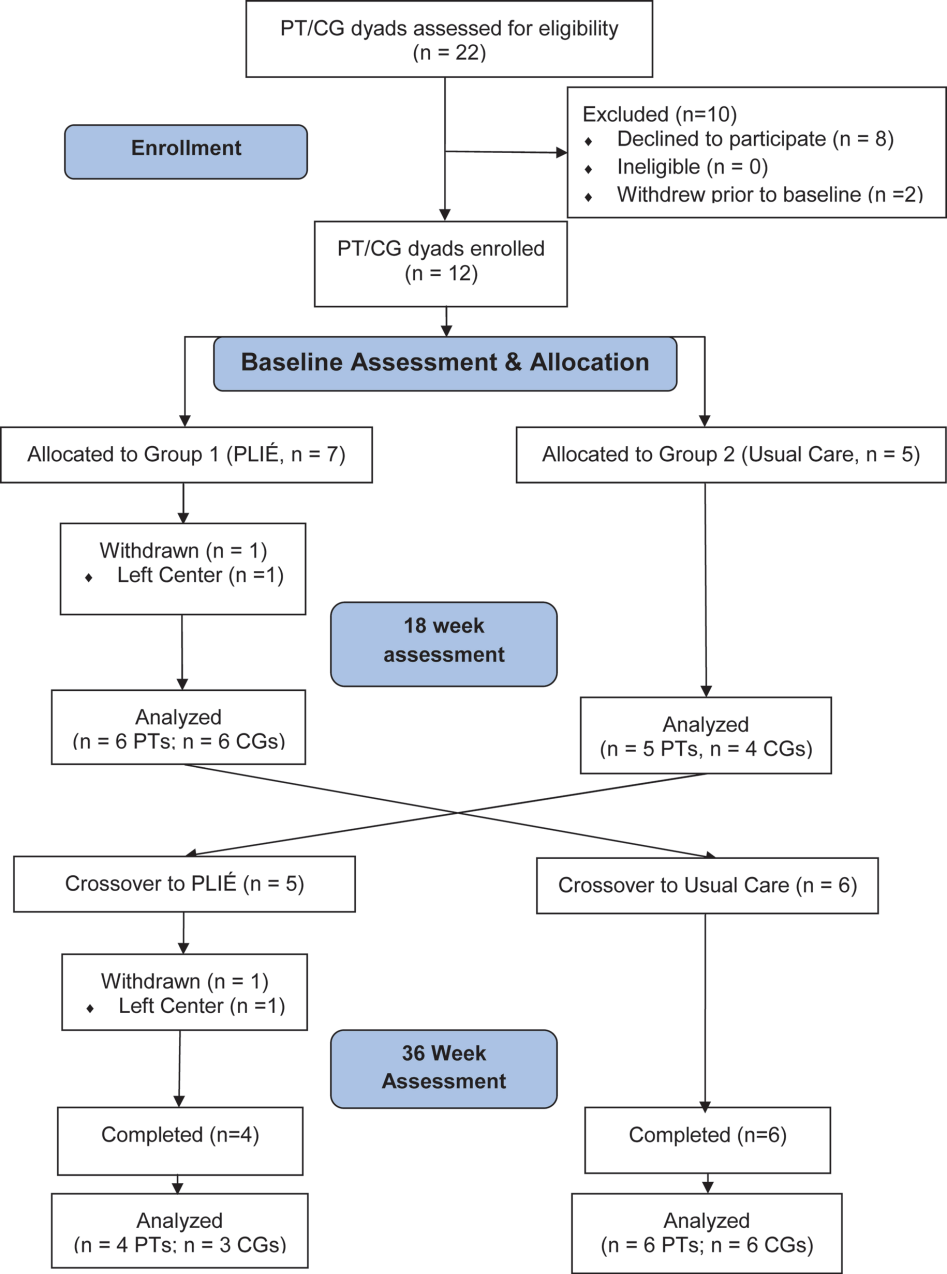
Flow Diagram of Study Participants. A total of 22 participant (PT)/caregiver (CG) dyads were assessed for eligibility, of whom 10 were excluded and 12 were enrolled and allocated to Group 1 (n = 7) or Group 2 (n = 5). Group 1 participated in the Preventing Loss of Independence through Exercise (PLIÉ) program while Group 2 participated in Usual Care activities from weeks 1 to 18. The groups then crossed over, and Group 1 returned to Usual Care activities while Group 2 participated in PLIÉ from weeks 19 to 36. Assessments were performed at baseline, 18 weeks and 36 weeks. One participant withdrew from Group 1 prior to the 18-week assessment and one participant withdrew from Group 2 prior to the 36-week assessment. In addition, one CG in Group 2 did not complete the 18- or 36-week assessments.

We pilot-tested PLIÉ by performing a 36-week cross-over study (Fig. 1) at a social adult day program for individuals with dementia in San Francisco, CA. Group 1 participated in the PLIÉ program at least 2 days per week for 45 minutes from weeks 1 to 18 while Group 2 engaged in usual activities (Usual Care, UC), which included standard chair-based exercises. From weeks 19 to 36, the groups crossed over, and Group 1 returned to usual activities while Group 2 participated in the PLIÉ program at least 2 days per week for 45 minutes. Standardized assessments were performed in all participants at baseline, week 18 (cross-over) and week 36 by trained research assistants who were blinded to group assignment.

While participants were receiving the PLIÉ intervention, exercise instructors also met with participants and caregivers together on a monthly basis for a total of up to 4 home visits, to provide targeted exercise instruction and better assess participants’ goals and interests. Caregivers in both groups were called on a biweekly basis to assess for adverse events throughout the study period.

### Eligibility and Enrollment

Participants and caregivers were enrolled as dyads. Inclusion criteria for participants were: age ≥ 55 years, a diagnosis of cognitive impairment or dementia of any type or severity, adult day program attendance at least 2 days/week, recommended by adult day staff, English language fluency and caregiver consent. These criteria were designed to be as inclusive as possible to reflect the real world heterogeneity of adult day programs. Most clients at this center had mild to moderate Alzheimer’s disease or vascular dementia. Exclusion criteria for participants were: lack of assent to study procedures. Inclusion criteria for caregivers were: current provision of care to primary participant and ability to answer questions about the primary participant’s physical function, behaviors, quality of life and their own level of stress. Exclusion criteria for caregivers were: major neurologic or psychiatric condition, life expectancy < 1 year, evidence of cognitive impairment or inability to consent to study procedures.

Potential participant/caregiver dyads were first contacted by staff at the adult day program. Those who agreed were then contacted by research staff and invited to participate in the study. As the day program already included daily exercise and required all clients to receive medical clearance prior to joining, our study did not require additional medical clearance.

### Setting

All intervention procedures took place at the adult day center or participant/caregiver homes. Outcome assessments for the participants were performed at the day center. Caregiver outcome assessments were performed at either the center or the caregiver’s home, based on their preference. Assessments were timed so that they did not overlap with intervention classes to ensure adequate blinding of assessors.

### PLIÉ Intervention

The PLIÉ program followed the Guiding Principles shown in Table 1 regarding the manner in which the program was taught. Briefly, the guiding principles included repetition with variation; progressive, functional movements; slow-pace and step-by-step instruction; participant-centered goal orientation; body awareness, mindfulness, and breathing; social interaction and positive emotions. In addition, each class followed a basic class structure that included greetings (5 minutes), body awareness warm-up (5 minutes), seated exercises (15 minutes), sit-to-stand and standing exercises (15 minutes) and closing/appreciations (5 minutes) (see S1 Appendix for additional details). The PLIÉ program was delivered for 45-minute sessions, 3 days/week throughout the study period. Exercise instructors had been previously certified in at least one body awareness-focused approach to exercise (e.g., Feldenkrais or Rosen method)[34,35] and were trained to administer the PLIÉ program using a detailed manual that was developed for this pilot study. Adherence to the protocol was monitored through class visits by the principal investigator and co-investigators; review of daily class reports and logs of the specific exercises taught at each class; and weekly team meetings.

### Usual Care (UC) Control

Participants in the UC group performed standard chair-based exercises in a separate room led by adult day center staff members for approximately 20 minutes followed by other group activities such as music and art appreciation. These exercises were designed to increase heart rate, strength and flexibility by engaging all major muscle groups, although heart rate was not routinely monitored. Key differences between PLIÉ versus UC exercises included: 1) sitting in a circle vs. sitting in rows facing instructor; 2) smaller group (up to 7 participants) vs. larger group (up to 30 participants); 3) repetition with variation vs. repetition with little variation; 4) progressive functional movement sequences vs. non-progressive movement; 5) slow pace vs. fast pace; 6) encouragement of social interaction between participants vs. encouragement of social interaction with instructor; 7) in-the-moment adaptation based on participants’ responses vs. routine delivery of class content; and 8) self-focus on mindful body awareness (interoception) vs. outward focus on copying the instructor’s movement (exteroception). Interoceptive versus exteroceptive focus distinguishes sensory attention towards perceptions of sensations from inside one’s own body, such as from movements and breathing,[36] from audio-visual attention towards a group leader. Center staff did not observe the PLIÉ classes taught by research staff.

### Measures

All outcome measures were selected because they are standard in the field and have well-established validity and reliability. Assessments were performed at baseline, 18 weeks (cross-over) and 36 weeks in both participants and caregivers. As the goal of the study was to estimate effect sizes for a larger study, we did not pre-specify primary or secondary outcomes but rather measured a range of different domains using standard measures. Exercise ‘dose’ was measured based on number of classes attended.

### Participant Measures

**Physical Performance**. Our primary measure of the physical effects of the program in participants was physical performance. This was assessed with the Short Physical Performance Battery (SPPB), which was developed by the National Institute on Aging to provide an objective tool for evaluating lower extremity functioning in older adults. The test includes repeated chair stands, tandem balance testing and 8’ walking speed.[37] A recent systematic review of instruments to measure physical performance in older adults concluded that the SPPB was one of the best tools available based on its reliability, validity and responsiveness.[38] Three additional items from the Senior Fitness Test (SFT) were added to assess flexibility (sit-and-reach, back scratch) and mobility (8-foot up-and-go).[39]

**Cognitive Function**. Cognitive function was assessed in participants with the Alzheimer’s Disease Assessment Scale—Cognitive Subscale (ADAS-cog),[40] which is one of the most commonly used primary outcome measures in dementia drug treatment trials. It is an 80-point scale that includes direct assessment of learning (word list), naming (objects), following commands, constructional praxis (figure copying), ideational praxis (mailing a letter), orientation (person, time, place), recognition memory and remembering test instructions. Prior studies have found the ADAS-cog to be valid and reliable with Cronbach’s alpha greater than 0.8 and test-retest reliability above 0.9.[41]

**Quality of Life**. Quality of life in participants was assessed with the Quality of Life Scale in Alzheimer’s Disease (QOL-AD),[42] which is a brief, 13-item measure that asks parallel questions about the affected individual’s quality of life from their perspective and the caregiver’s perspective. Scores may range from 13–52 points. Prior studies have found that the QOL-AD is a valid and reliable measure, with Cronbach’s alpha of 0.84 for patient reports and 0.86 for caregiver reports and interrater reliability based on Cohen’s kappa values >0.70.[43]

### Caregiver Measures

Caregiver measures included questionnaires about the participant’s functional status, quality of life and dementia-related behaviors as well as their own level of distress with behaviors and overall burden.

**Participant Function**. Participant’s physical function was assessed with the Alzheimer’s Disease Cooperative Study—Activities of Daily Living (ADCS-ADL) scale.[44] The ADCS-ADL is a 78-point scale that assesses functional ability in 23 daily activities based on informant report. It is a standard measure for assessment of functional status in dementia drug treatment trials, with 2-month test-retest reliability based on kappa values for individual items of 0.4–0.75.[44]

**Participant Behavior**. Participant's dementia-related behaviors were assessed with the Neuropsychiatric Inventory (NPI), [45] which is a 144-point informant-based questionnaire that assesses 12 behavioral domains common in dementia including frequency, severity and impact on caregiver distress. There are two summary scores: a frequency*severity total (NPI-FS) and caregiver distress total (NPI-CD). The NPI has good test-retest reliability (0.79–0.86) and good internal consistency (Cronbach’s alpha, 0.87–0.88).[45]

**Participant Quality of Life**. Participant's quality of life was assessed with the QOL-AD which, as noted above, asks similar questions of both the individual and the caregiver,[42] and has established validity and reliability.[43]

**Caregiver Burden**. Caregiver burden was assessed with the Caregiver Burden Inventory (CBI), which is a 96-point scale that includes 24 items and 5 domains.[46] Caregivers are asked to rate how often each statement describes their feelings (never, rarely, sometimes, quite frequently, nearly always). The total score may range from 0 to 96 with higher scores reflecting greater feelings of burden. The CBI has good internal consistency (Cronbach’s alpha, 0.73–0.86_ENREF_39).[46]

### Other Measures

Demographic information (age, sex, race, education) was determined for both participants and caregivers based on caregiver report. Caregivers also were asked about the participant’s diagnosis (Alzheimer’s disease, vascular dementia, other/unknown), the number of years they had been providing care, their relationship with the participant (spouse, child, other) and their marital status. The Modified Mini-Mental State Examination (3MS)[32] was performed at baseline only to provide a measure of global cognitive function. The 3MS is similar to the original Mini-Mental State Exam (MMSE)[33] but includes an expanded scoring system (range: 0 to 100 points) and some additional items. Prior studies have found that the 3MS has high internal consistency (alpha = 0.87) and is more sensitive than the MMSE for detecting dementia.[47]

### Group Assignment and Blinding

Due to the limited number of potential participants attending on a given day, it was not possible to randomize. Instead, the Principal Investigator assigned participants to groups based on their days of attendance at the day program (Group 1: Monday, Tuesday, Thursday; Group 2: Monday, Wednesday, Thursday) and to balance genders. Research assistants who collected outcome data were blinded to group assignment.

### Data Analysis

Characteristics of Group 1 and Group 2 were compared using *t*-tests for continuous variables and Chi-square tests for categorical variables. Means were calculated for all outcome measures at each of the three time points (baseline, 18 weeks, 36 weeks) by group (PLIÉ vs. UC). Our primary outcome was the ‘between-group’ effect size from baseline to 18 weeks, which was defined as the change in Group 1 (PLIÉ) minus change in Group 2 (UC) divided by the pooled baseline standard deviation (SD). Signs were reversed for measures in which lower scores reflected better outcomes, so that positive values indicate greater improvement with PLIÉ and negative values reflect greater improvement with UC. Only those who completed assessments at both time points were included in calculations. An effect size of ≥ 0.25 SDs was defined as ‘clinically meaningful’ based on prior studies of effect sizes for current dementia medications.[5]

Although there are no well-accepted criteria for defining an effect size as clinically meaningful, an effect size ≥0.20 is generally considered small, while an effect size ≥0.50 would be considered medium and an effect size ≥0.80 is considered large.[48] To capitalize on the cross-over design, we also calculated ‘within-group’ effect sizes for both groups, which were defined as change during PLIÉ minus change during UC divided by baseline SD. Thus, for Group 1 (PLIÉ first), the within-group effect size was calculated as change from baseline to 18 weeks minus change from 18 to 36 weeks divided by baseline SD, whereas for Group 2 (UC first), the within-group effect size was calculated as change from 18 to 36 weeks minus change from baseline to 18 weeks divided by baseline SD.

## Results

The flow of participants through the study is shown in Fig. 1. Twenty-two individuals were assessed for eligibility from 10/3/11 to 1/25/12. Eight declined to participate, and two withdrew prior to the baseline assessment. Twelve participants were enrolled in the study—seven of whom were assigned to Group 1 and five to Group 2. One participant in Group 1 withdrew prior to the 18-week assessment due to general dissatisfaction with the adult day program, and one participant in Group 2 withdrew prior to the 36-week assessment due to placement in a residential facility. Group 1 participated in the PLIÉ program from 11/14/11 to 3/29/12 and then returned to usual activities, while Group 2 started with usual activities and then participated in PLIÉ from 4/2/12 to 8/23/12. The mean ± SD (range) number of PLIÉ classes attended was 39 ± 4 (34–46) in Group 1 and 39 ± 9 (30–47) in Group 2. Eleven participants completed the 18-week assessment and were included in between-group effect size calculations for participant measures (6 PLIÉ, 5 UC). Ten caregivers completed the 18-week assessment and were included in between-group effect size calculations for caregiver measures (6 PLIÉ, 4 UC). Ten participants and nine caregivers completed the 36-week assessments.

Participants had a mean age of 84 ± 4 years while caregivers had a mean age of 56 ± 13 years (Table 2). Most participants were white, female and had high levels of education; mean (SD) 3MS scores were 60.9 (18.9) at baseline, which is consistent with mild to moderate dementia. Most caregivers were married daughters who had provided care for an average of 3.6 years. There were no significant differences in either participant or caregiver measures between groups at baseline.

**Table 2:** Baseline Characteristics.

Characteristic*	Group 1 (n = 6)	Group 2 (n = 5)
Participant		
Age, years	85.67 ± 5.61	81.60 ± 2.30
Gender, female	5 (83.33%)	4 (80.00%)
Education, years	13.00 ± 3.03	15.60 ± 2.97
Race, white	5 (83.33%)	4 (80.00%)
3MS Baseline Score	61.83 ± 15.83	59.80 ± 22.55
Diagnosis, Alzheimer’s	4 (66.67%)	2 (40.00%)
Diagnosis, Vascular Dementia	1 (16.67%)	2 (40.00%)
Diagnosis, Other/DK	1 (16.67%)	1 (20.00%)
Caregiver		
Age, years	57.50 ± 14.47	54.60 ± 11.06
Gender, female	5 (83.33%)	4 (80.00%)
Education, years	16.17 ± 2.56	18.40 ± 1.67
Providing care, years†	4.33 ± 2.94	2.50 ± 1.29
Relationship, Daughter/Son	5 (83.33%)	4 (80.00%)
Relationship, Spouse	1 (16.67%)	1 (20.00%)
Marital Status, Married/Partnered	4 (66.67%)	4 (80.00%)
Marital Status, Divorced/Single	2 (33.33%)	1 (20.00%)

*Mean ± SD for continuous variables, and N (%) for categorical variables.

†One caregiver from the ‘usual care’ group did not provide this information.

Mean scores at baseline, 18-week change and between-group effect size estimates for participant measures are shown in Table 3. Physical performance scores improved 1.0 point (5.2 to 6.2) over 18 weeks in the PLIÉ group compared to 0.2 points (5.4 to 5.6) in the UC group, for an effect size of +0.34 SDs. Additionally, cognitive function scores improved 4.7 points (27.1 to 22.4, with lower scores reflecting better cognitive function) over 18 weeks in the PLIÉ group compared to a worsening of 2.4 points (23.7 to 26.1) in the UC group, for an effect size of +0.76 SDs. Self-reported quality of life scores improved 6.0 points (40.5 to 46.5) over 18 weeks in the PLIÉ group compared to 2.6 points (40.4 to 43.0) in the UC group, for an effect size of +0.83 SDs. There also was evidence of greater improvement with PLIÉ than UC on the back scratch (+0.35 SDs) and 8 foot up & go (+0.24 SDs) but worsening on the sit & reach measure (-0.32 SDs).

**Table 3:** Between-Group Effect Sizes in Participant Measures*, Baseline to 18 Weeks.

Measure	Time	Group 1 (PLIÉ, n = 6)	Group 2 (UC, n = 5)	Effect Size**
Physical performance (SPPB)^a^	Baseline	5.17 (2.99)	5.40 (1.67)	
	18-Week Change	1.00 (2.68)	0.20 (1.64)	**+ 0.34**
Cognitive function (ADAS-cog)^b^	Baseline	27.06 (8.43)	23.73 (10.78)	
	18-Week Change	-4.61 (6.37)	2.40 (3.42)	**+ 0.76**
Quality of life (QOL-AD)^a^	Baseline	40.50 (3.94)	40.40 (4.72)	
	18-Week Change	6.00 (6.20)	2.60 (5.50)	**+ 0.83**
SFT—back scratch^a^	Baseline	-5.50 (4.14)	-9.0 (3.16)	
	18-Week Change	1.58 (1.15)	0.20 (3.65)	**+ 0.35**
SFT—sit & reach^a^	Baseline	-0.17 (3.83)	-1.7 (4.99)	
	18-Week Change	-1.05 (2.39)	0.30 (3.96)	- 0.32
SFT—8-foot up & go^b^	Baseline	14.81 (3.63)	15.27 (6.61)	
	18-Week Change	-2.23 (3.54)	-1.03 (2.37)	+ 0.24

SPPB, Short Physical Performance Battery; ADAS-cog (Alzheimer’s Disease Assessment Scale—cognitive subscale; QOL-AD, Quality of Life in Alzheimer’s Disease; SFT, Senior Fitness Test.

a: higher scores better;

b: lower scores better.

*Means (SD).

**Effect size calculated by subtracting mean change in Group 1 from mean change in Group 2 and dividing by the pooled baseline standard deviation; + values favor PLIÉ, and − values favor Usual Care. Bolded effect sizes favor PLIÉ and were ≥ 0.25. Data missing as follows: SFT back scratch (group 1, n = 1, both time points).

Mean scores at baseline, 18-week change and between-group effect size estimates for caregiver measures are shown in Table 4. Caregiver ratings of participants’ quality of life improved 2.2 points (36.3 to 38.5) in the PLIÉ group compared to no change (30.0 to 30.0) in the UC group (effect size, +0.33 SDs). The frequency and severity of dementia-related behaviors declined in both groups (PLIÉ: 9.7 to 6.3; UC: 14.5 to 11.5) with no evidence of difference between groups (effect size, +0.025); however, caregiver distress related to dementia behaviors declined 1.7 points with PLIÉ (6.3 to 4.0) and increased 0.5 points with UC (8.5 to 9.0) for an effect size of +0.28 SDs. Caregiver burden also declined 5.5 points with PLIÉ (29.8 to 24.3 points) and increased 1.7 points with UC (32.5 to 34.2) for an effect size of +0.49 SDs. There was little evidence of change in caregiver-reported participant function with either PLIÉ (48.8 to 48.3) or UC (47.2 to 47.8) (effect size, -0.07 SDs).

**Table 4:** Between-Group Change in Caregiver Measures*, Baseline to 18 Weeks.

Measure	Time	Group 1 (PLIÉ, n = 6)	Group 2 (UC, n = 4)	Effect Size**
Participant function (ADCS-ADL)^a^	Baseline	48.83 (10.26)	47.25 (20.16)	
	18-Week Change	-0.50 (9.85)	0.50 (2.65)	- 0.07
Participant quality of life (QOL-AD)^a^	Baseline	36.33 (5.99)	30.00 (6.32)	
	18-Week Change	2.17 (5.00)	0.00 (3.56)	**+ 0.33**
Participant behaviors (NPI-FS)^b^	Baseline	9.67 (14.72)	14.50 (13.58)	
	18-Week Change	-3.33 (9.11)	-3.00 (2.45)	+ 0.02
Participant behaviors (NPI-CD)^b^	Baseline	6.33 (12.23)	8.50 (7.23)	
	18-Week Change	-2.33 (8.87)	0.50 (3.70)	+ 0.21
Caregiver burden (CBI)^b^	Baseline	29.83 (12.80)	32.50 (19.50)	
	18-Week Change	-5.50 (3.21)	1.75 (11.62)	+ 0.49

ADCS-ADL, Alzheimer’s Disease Cooperative Study—Activities of Daily Living scale; QOL-AD, Quality of Life in Alzheimer’s Disease; NPI-FS, Neuropsychiatric Inventory—frequency*severity subscale; NPI-CD, Neuropsychiatric Inventory—caregiver distress subscale; CBI, Caregiver Burden Inventory.

a: higher scores better;

b: lower scores better.

*Means (SD).

**Effect size calculated by subtracting mean change in Group 1 from mean change in Group 2 and dividing by the pooled baseline standard deviation; + values favor PLIÉ, and − values favor Usual Care. Bolded effect sizes favor PLIÉ and were ≥ 0.25.

Table 5 provides within-group effect size estimates for both participant and caregiver measures for Group 1. One interpretation of the results is that there was evidence of greater improvement when participants were involved with PLIÉ (0 to 18 weeks) versus UC (19 to 36) for 9 of the 11 measures, including all of the measures that improved based on between-group effect size estimates as well as caregiver-reported frequency and severity of dementia-related behaviors (effect size, +0.59). An alternative interpretation is that the improvements observed with PLIÉ were maintained or continued to improve for several measures following cross-over to usual care. For example, physical performance scores improved 1 point from baseline to 18 weeks and an additional 0.33 points from 18 to 36 weeks, suggesting continued improvement. Similar trends were observed with cognitive function (additional 1.11 point improvement from 18 to 36 weeks) and 8 foot up & go (additional 1.21 second improvement). Conversely, quality of life declined following return to usual care from the perspective of both participants (-4.0 points) and caregivers (-0.33 points) while increases were observed in the frequency and severity of participant behaviors (+2.00 points) and caregiver burden (+0.67 points).

**Table 5:** Within-Group Changes*and Effect Sizes in Participant and Caregiver Measures, Group 1.

Measure	0 to 18 week change (PLIÉ)	19 to 36 week change (UC)	Effect Size**
Participant	N = 6	N = 6	
Physical performance (SPPB)^a^	1.00 (2.68)	0.33 (0.82)	**+ 0.25**
Cognitive function (ADAS-cog) ^b^	-4.61 (6.37)	-1.11 (1.78)	**+ 0.55**
Quality of life (QOL-AD) ^a^	6.00 (6.20)	-4.00 (4.20)	**+ 1.61**
SFT—Back scratch ^a^	1.58 (1.15)	-0.78 (1.15)	**+ 0.99**
SFT—Sit and reach ^a^	-1.05 (2.39)	0.13 (2.34)	- 0.49
SFT—8 foot up and go ^b^	-2.23 (3.54)	-1.21 (2.34)	**+ 0.29**
**Caregiver**	**N = 6**	**N = 6**	
Participant function (ADCS-ADL) ^a^	-0.50 (9.85)	0.67 (3.88)	- 0.12
Participant QOL (QOL-AD) ^a^	2.17 (5.00)	-0.33 (3.56)	**+ 0.50**
Participant behaviors (NPI-FS) ^b^	-3.33 (9.11)	2.00 (4.80)	**+ 0.59**
Participant behaviors (NPI-CD) ^b^	-2.33 (8.87)	0.00 (2.10)	**+ 0.26**
Caregiver burden (CBI) ^b^	-5.50 (3.21)	0.67 (6.15)	**+1.92**

SPPB, Short Physical Performance Battery; ADAS-cog, Alzheimer’s Disease Assessment Scale—cognitive subscale; QOL-AD, Quality of Life in Alzheimer’s Disease scale; SFT, Senior Fitness Test; ADCS-ADL, Alzheimer’s Disease Cooperative Study—Activities of Daily Living scale; NPI-FS, Neuropsychiatric Inventory—frequency*severity subscale; NPI-CD, Neuropsychiatric Inventory—caregiver distress subscale; CBI, Caregiver Burden Inventory.

a: higher scores better;

b: lower scores better.

*Means (SD).

**Effect size calculated by subtracting mean change from 19 to 36 weeks from mean change from 0 to 18 weeks and dividing by the baseline standard deviation; + values favor PLIÉ, and − values favor Usual Care. Bolded effect sizes favor PLIÉ and were ≥ 0.25. Data missing as follows: SFT back scratch (n = 1, both time points) SFT—8 foot up and go (n = 1, 0 to 18 weeks), NPI-FS (n = 1, 19 to 36 weeks).

Table 6 provides within-group effect size estimates for both participant and caregiver measures in Group 2. Consistent with between-group analyses and within-group analyses in Group 1, there was evidence of greater improvement when participants were involved with PLIÉ (19 to 36 weeks) versus UC (0 to 18 weeks) for measures of physical performance (+0.34 SDs), cognitive function (+0.38 SDs), and caregiver ratings of participant quality of life (+0.47 SDs) as well as sit & reach (+0.71 SDs), 8 foot up & go (+0.32 SDs) and caregiver distress related to participant behaviors (+0.49 SDs). In contrast, there was evidence of greater improvement with UC than PLIÉ for participant-rated quality of life (-1.06 SDs), back scratch (-0.40 SDs), participant function (-0.31 SDs), and the frequency and severity of participant behaviors (-1.22 SDs) and no evidence of difference for caregiver burden (-0.05 SDs).

**Table 6:** Within-Group Changes* and Effect Sizes in Participant and Caregiver Measures, Group 2.

Measure	0 to 18 week change (UC)	19 to 36 week change (PLIÉ)	Effect Size**
Participant	N = 5	N = 4	
Physical performance (SPPB)^a^	0.20 (1.64)	0.75 (1.89)	**+ 0.34**
Cognitive function (ADAS-cog) ^b^	2.40 (3.42)	1.09 (4.31)	**+ 0.38**
Quality of life (QOL-AD)^a^	2.60 (5.50)	-3.25 (2.63)	- 1.06
SFT—Back scratch^a^	0.20 (3.65)	-1.25 (3.62)	- 0.40
SFT—Sit and reach^a^	0.30 (3.96)	3.13 (1.11)	**+ 0.71**
SFT—8 foot up and go^b^	-1.03 (2.37)	-1.80 (3.33)	**+ 0.32**
**Caregiver**	**N = 4**	**N = 3**	
Participant function (ADCS-ADL) ^a^	0.50 (2.65)	-0.33 (2.08)	- 0.31
Participant QOL (QOL-AD) ^a^	0.00 (3.56)	1.67 (1.53)	**+ 0.47**
Participant behaviors (NPI-FS) ^b^	-3.00 (2.45)	0 (25.51)	- 1.22
Participant behaviors (NPI-CD) ^b^	0.50 (3.70)	-1.33 (4.04)	**+ 0.49**
Caregiver burden (CBI)^b^	1.75 (11.62)	2.33 (2.31)	- 0.05

SPPB, Short Physical Performance Battery; ADAS-cog, Alzheimer’s Disease Assessment Scale—cognitive subscale; QOL-AD, Quality of Life in Alzheimer’s Disease scale; SFT, Senior Fitness Test; ADCS-ADL, Alzheimer’s Disease Cooperative Study—Activities of Daily Living scale; NPI-FS, Neuropsychiatric Inventory—frequency*severity subscale; NPI-CD, Neuropsychiatric Inventory—caregiver distress subscale; CBI, Caregiver Burden Inventory.

a: higher scores better;

b: lower scores better.

*Means (SD).

**Effect sizes calculated by subtracting mean change 19 to 36 weeks from mean change 0 to 18 weeks and dividing by the baseline standard deviation. + values favor PLIÉ, and − values favor Usual Care. Bolded effect sizes favor PLIÉ and were ≥ 0.25.

Five adverse events occurred during or shortly after participating in the PLIÉ program and were classified as possibly study-related including dizziness/nausea (n = 1), legs buckling later in the day (n = 1), falling forward on hands and knees during class (n = 1), and hip pain (n = 2). None were considered serious or unexpected or resulted in withdrawal from the study, and all resolved without affecting future class attendance.

## Discussion

The results of this pilot study suggest that PLIÉ may be associated with improvements in a wide range of outcomes with clinically meaningful between-group effect sizes for physical performance (0.34 SDs), cognitive function (0.76 SDs) and quality of life (0.83 SDs) in individuals with mild to moderate dementia as well as reduced caregiver burden (0.49 SDs) when compared with a usual care program that involved daily chair-based exercises.

The magnitude of improvement observed with PLIÉ is substantially larger than what has been observed with currently approved dementia medications such as cholinesterase inhibitors and memantine and affects a broader range of outcomes.[49] For example, a meta-analysis found that the median standardized effect sizes for cholinesterase inhibitors on cognitive function (measured using the ADAS-cog) were 0.15 for low doses, 0.23 for medium doses and 0.28 for high doses.[5] In addition, many patients choose to discontinue treatment with dementia medications due to substantial side effects such as diarrhea, vomiting, nausea and fatigue.[50] Memantine is approved for moderate to severe dementia only and is also associated with small improvements with effect sizes of 0.33 for cognitive function, 0.22 for behaviors, and 0.11 for function.[6] Furthermore, these medications do not alter disease progression[1] and have negligible effects on other measures including physical performance, quality of life and caregiver burden,[51] which in the present study showed evidence of clinically meaningful improvement with PLIÉ.

Several aspects of PLIÉ are unique and may have contributed to our findings of improved physical performance. The same sequence of events was repeated in each class, providing a structure that became familiar over time and was designed to promote procedural learning. Therefore, even when participants did not recall having participated in the class before, their bodies appeared to remember which movements came next in the sequence. In addition, the specific movements of PLIÉ were selected for their relationship to important daily functional activities such as being able to stand safely from a seated position. These functional movements were broken down into their component elements and slowly increased in difficulty over the course of the program. Instructors also motivated participants by relating the movements to participant’s individual interests and goals and by engaging participants in interactive group movement activities. This may have enabled participants to slowly build their capacity to perform more complex movements with ease over time and provided the movements with greater meaning.[52]

Other aspects of the program may have contributed to our findings of improved cognitive function and quality of life. During periods of rest, participants were encouraged to notice their breathing and how they felt, both physically and emotionally. This focus on mindful, in-the-moment body awareness may have had a calming effect on the mind, which could have resulted in greater attentional capacity and increases in cognitive function. In addition, the PLIÉ program explicitly focused on creating a warm, loving, non-judgmental environment, and some participants appeared to develop deeper social bonds with each other over the course of the program, which may have enhanced general feelings of well-being and quality of life.[52]

We also observed improvements in caregiver ratings of participants’ quality of life as well as their own levels of burden and distress. It is not clear whether these findings are related to the effects of exercises taught directly to the participants during the classes or the effects of the monthly home visits, when instructors demonstrated some of the exercises to caregivers and also provided caregiving advice based on their observations in the home environment.

Our pilot study has several important strengths. First, we compared PLIÉ with standard chair-based exercises, which are common in adult day settings. This provided greater context for the magnitude of improvement observed and enabled more accurate calculation of the sample size that would be required to perform a full-scale study. Second, we utilized a cross-over design, which enabled us to calculate both between-group and within-group effect sizes and to determine whether the effects seen with PLIÉ were maintained over an additional 18 weeks of follow-up. Third, we designed the study to methodologically mimic a drug study by including measures that are commonly used in dementia medication trials, which enabled comparison of the magnitude of our results to currently available dementia medications.

Several important limitations also should be considered. Most importantly, our sample size was not large enough to detect statistically significant effects of the intervention. However, our results provide data to calculate sample sizes for a larger trial. In addition, we were unable to randomize subjects to groups. However, the groups were comparable at baseline, and individuals who collected outcome data were blinded. Finally, we did not observe evidence of change in activities of daily living in either the PLIÉ or UC group. It is possible that a longer intervention would be required to document change in this domain.

In conclusion, out pilot study results suggest that PLIÉ—a novel, integrative exercise program that incorporates elements of conventional and complementary or integrative exercise modalities—may improve physical performance, cognitive function, and quality of life in individuals with mild to moderate dementia and may also reduce caregiver burden. Larger clinical trials of the PLIÉ program are warranted.

## Supporting Information

S1 CONSORT Checklist. (DOC)

S1 Appendix Overview of the Preventing Loss of Independence through Exercise (PLIÉ) program and basic class structure. (DOCX)

S1 Protocol Originally approved trial protocol. (PDF)
