# Supplementary material for: Preventing Loss of Independence through Exercise (PLIÉ): A Pilot Clinical Trial in Older Adults with Dementia
Source: PLoS One. 2015 Feb 11;10(2):e0113367. doi: 10.1371/journal.pone.0113367 (PMC4324943; doi:10.1371/journal.pone.0113367)
Supplement: S1 Appendix — (DOCX) [file pone.0113367.s002.docx]

**APPENDIX S1:**

**A Brief Overview of the Preventing Loss of Independence through Exercise (PLIÉ) Program**

***Background***

The Preventing Loss of Independence through Exercise (PLIÉ) program was developed based on recent discoveries in neuroscience and experimental psychology, which have found that, although explicit memory (conscious learning of new information) is impaired in individuals with dementia, implicit memory (learning that occurs without conscious awareness) is relatively preserved. This is particularly true of implicit memory that involves perceptual or motor learning rather than conceptual learning, including procedural learning (learning to perform procedures).

We hypothesized that an exercise program that focused on training procedural memory to build the strength and capacity to perform the movements that are most needed for daily function (e.g., transitioning safely from sitting to standing) would help individuals with dementia to maintain functional independence. In addition, we hypothesized that the benefits of the program would be enhanced by combining or integrating ‘best elements’ from both conventional and complementary/alternative exercise approaches, particularly through greater in-the-moment body awareness and social connection. We named this integrative exercise program Preventing Loss of Independence through Exercise (PLIÉ).

We developed the PLIÉ program by observing local group exercise classes for individuals with dementia and by consulting with experts from a wide range of exercise approaches (including physical therapy, occupational therapy, yoga, tai chi, Feldenkrais, Rosen method, and dance movement therapy). We then convened an all-day video conference with representatives from each of these approaches (academic and non-academic; local, national and international) with the goals of: 1) having each expert demonstrate key aspects of their approach to working with individuals with dementia, focusing on movements to maintain functional status; 2) discussing as a group the similarities and differences between the approaches and 3) identifying key elements from each approach as well as guiding principles that were common across multiple approaches. Table 1 provides a summary of the Guiding Principles of the program. A list of individuals who provided input into program development is provided in the Acknowledgements.

***PLIÉ Basic Class Structure***

PLIÉ involves repetition of standardized sequences of movements/events at each class. The specific exercise sequences selected each day may be varied based on the needs, ability levels, interests and in-the-moment responses of the group. However, certain movements were always performed to give the class a consistent structure and to promote procedural learning. Movement sequences are designed to build progressively over time to slowly increase functional movement.

1. ***Greetings (5 minutes).*** Goal: to convey that everyone present is individually recognized and welcomed into a social group and create a positive, welcoming group environment where everyone shares the same purpose, which builds the foundation for social support. Each class begins with the participants and the instructor sitting in chairs in a circle. To ensure the safety of participants, there are no more than 6 individuals per instructor. The instructor begins by greeting each participant by name and making eye contact.
2. ***Body awareness warm-up (5 minutes).*** Goal: to increase somatic awareness (i.e., awareness of the body) and stimulate flow of blood and oxygen throughout the body. A central hypothesis of the PLIÉ program is that movement performed with somatic awareness can result in improved function. Every class begins with a body awareness warm-up. Class participants are invited to massage, rub, pat and notice their bodies, starting with their hands and including arms, legs, hips, belly, back, neck and face. Participants then are invited to inhale deeply as they raise their arms overhead and make a sound as they exhale and lower their arms. Once these movements become familiar, group members are invited to initiate movements and sounds of their choice. After these exercises, participants are invited to take a mindful rest, sitting quietly with eyes open or closed, allowing time to notice any changes in their bodies.
3. ***Seated exercises (15 minutes).*** Goal: to present exercises in a graded fashion using repetition to promote procedural and functional learning. A series of seated exercises are performed to prepare the body for activities such as standing and walking and to extend range of motion. These exercises build progressively over the course of the class, beginning with simple movements and increasing in complexity. Standardized variations are incorporated to provide variety, as participants’ familiarity and facility with the movements increases. While performing exercises, class participants are invited to notice sensations as well as things around them (e.g., paintings on the wall, weather outside) and, if they wish, to share their in-the-moment observations with the group. Some exercises are designed to encourage social interaction by having participants reach out to their neighbor or across the circle to touch hands or elbows. Mindful rests between movements also allow time for participants to continue to bring attention back to their somatic awareness. Once simple exercises appear to be performed with greater ease by participants, exercises combine upper and lower body movements to support more complex functions. Brief musical selections (4-6 minutes) based on participant or caregiver suggestions are incorporated once or twice a week at the discretion of exercise instructors to facilitate movement and social interaction.
4. ***Sit-to-Stand and Standing exercises (15 minutes).*** Goal: to teach key elements for transitioning from sitting to standing. These exercises progress through the movements required to transition safely from sitting to standing in a stepwise fashion: feeling feet flat on the floor, scooting forward in chair, leaning head forward, slowly lifting buttocks off chair a few times and returning to sitting, and finally standing upright. Instructors may provide gentle assistance with standing or may place a walker in front of participants if there are concerns about balance. Participants also may choose to remain seated throughout these exercises or to sit down while others continue to stand. Participants are allowed to engage and disengage in activities as they wish and are allowed to rest as needed. Those who choose to stand perform a series of balancing exercises that focus on shifting weight and moving together as a group. After the balancing exercises have been completed, all participants return to sitting and are invited to take a mindful rest, breathe deeply and feel their bodies.
5. ***Closing & Appreciations (5 minutes).*** Goal: to consolidate what was learned during class by linking exercises with positive somatic and emotional experiences. These exercises bring participants’ attention back into their own bodies and focus on body awareness. The body rub and full-body breathing are repeated, ending with a mindful rest. Finally, participants are given an opportunity to share appreciations with the group.
