# Supplementary material for: Preventing Loss of Independence through Exercise (PLIÉ): A Pilot Clinical Trial in Older Adults with Dementia
Source: PLoS One. 2015 Feb 11;10(2):e0113367. doi: 10.1371/journal.pone.0113367 (PMC4324943; doi:10.1371/journal.pone.0113367)
Supplement: S1 Protocol — (PDF) [file pone.0113367.s003.pdf]

1.0 General Information

1.1 *Enter the full title of your study:

Preventing Loss of Independence through Exercise (PLIÉ)

1.2 *Enter the study number or study alias

PLIÉ Project

2.0 Add Department(s)

2.1 List departments and/or research programs associated with this study

Primary Dept?	Department Name
●	**UCSF** - *101001* - M_Osher Center
○	**UCSF** - *133144* - M_Psych-LPPI-Core-General

3.0 Assign key project personnel(KSP) access to the project

3.1 *Please add a Principal Investigator for the project:

Deborah E Barnes

Select if applicable
- ☐ Department Chair
- ☐ Fellow

3.2 If applicable, please select the Research Staff personnel:

A) Additional Investigators

- ☐ Margaret A Chesney
  Co-Principal Investigator
- ☐ Wolf E Mehling
  Other Investigator
- ☐ Kristine Yaffe
  Other Investigator

B) Research Support Staff

- Wendy Santos-Modesitt
  Research Assistant
- Eveline Wu
  Research Assistant

3.3 ***Please add a Project Contact:**

01. Deborah E Barnes

11. Wendy Santos-Modesitt

The Project Contact(s) will receive all important system notifications along with the Principal Investigator. (e.g. The project contact(s) are typically either the Study Coordinator or the Principal Investigator themselves).

3.4 If applicable, please add a Faculty Advisor/Mentor:

3.5 If applicable, please select the Designated Department Approval(s):

Add the name of the individual authorized to approve and sign off on this protocol from your Department (e.g. the Department Chair or Dean).

4.0 Qualifications of Key Study Personnel

4.1 List the study responsibilities and qualifications of any individuals who qualify as Key Study Personnel (KSP) at UCSF and affiliated sites ONLY by clicking the "Add a new row" button:

NOTE: This information is required and your application will be considered incomplete without it.

KSP Name	Description of Study Responsibilities	Qualifications
Dr. Barnes, Deborah E, PhD	As PI, Dr. Barnes will oversee all aspects of study.	Dr. Barnes is an Assoc. Prof of Psychiatry at UCSF. Her research focuses on identifying risk factors for cognitive impairment and dementia and conducting clinical trials of strategies to enhance cognitive and physical function in older adults.

Chesney, Margaret A, PhD	As Co-PI, Dr. Chesney will provide senior level guidance, particularly regarding aspects of the project related to integrative medicine.	Dr. Chesney is Director of the Osher Center for Integrative Medicine. She was the interim director of the National Institute for Complementary and Integrative Medicine at the NIH and involved in its research.
Mehling, Wolf E	As Co-Investigator, Dr. Mehling will work with Dr. Barnes in the development and implementation of the study, particularly aspects of the study related to integrative medicine.	Dr. Mehling is an Assoc. Prof. of Clinical Family and Community Medicine and on faculty at the Osher Center for Integrative Medicine. His research focuses on body-oriented complementary therapies and mind-body interactions.
Dr. Yaffe, Kristine MD	As Co-Investigator, Dr. Yaffe will provide guidance on development and implementation of the study, particularly aspects of the study related to working with individuals with dementia.	Dr. Yaffe is a Professor of Psychiatry, Neurology and Epidemiology/Biostatistics at UCSF. Her research focuses on identification of potentially modiable risk factors for cognitive impairment and dementia.
Santos-Modesitt, Wendy	As a Research Assistant, Ms. Santos-Modesitt will assist with enrolling, scheduling, consenting and testing research subjects.	Ms. Santos-Modesitt is a graduate student in clinical psychology at Alliant University. She has been working with Dr. Barnes for more than a year and has experience working with individuals with dementia.
Wu, Eveline	Ms. Wu will be responsible for leading the exercise intervention with oversight from Drs. Barnes and Mehling.	Ms. Wu is a certified Feldenkrais instructor with more than 10 years of experience working with a wide range of clients including older adults and special needs children.

5.0 Initial Screening Questions

5.1 * This study involves human stem cells (including iPS cells and adult stem cells), gametes or embryos:

- ● No
- ○ Yes, and requires CHR and GESCR review
- ○ Yes, and requires GESCR review, but NOT CHR review

5.2 * **This application involves a Humanitarian Use Device:**

- ● No
- ○ Yes, and it includes a research component
- ○ Yes, and it involves clinical care ONLY

5.3 * **This is a CIRB study (e.g. the NCI CIRB will be the IRB of record):**

○ Yes ● No

5.4 * **This application includes a request to rely on another UC IRB to be the IRB of record:**

○ Yes ● No

Note: If this request is approved, the CHR will **NOT** review and approve this study. Another UC campus will be the IRB of record.

6.0 Application Type

6.1 * **This research involves:**

- ● Minimal risk
- ○ Greater than minimal risk

6.2 * **This application is:**

- ○ Full Committee
- ● Expedited
- ○ Exempt

6.3 **If you think this study qualifies for expedited review, select the regulatory category(ies) that the research falls under:**

- □ Category 1: A very limited number of studies of approved drugs and devices
- □ Category 2: Blood sampling
- □ Category 3: Noninvasive specimen collection (e.g. buccal swabs, urine, hair and nail clippings, etc.)
- □ Category 4: Noninvasive clinical procedures (e.g. physical sensors such as pulse oximeters, MRI, EKG, EEG, ultrasound, moderate exercise testing, etc.)
- □ Category 5: Research involving materials (data, documents, records, or specimens) that were previously collected for either nonresearch or research purposes
- □ Category 6: Use of recordings (voice, video, digital or image)
- ■ Category 7: Low risk behavioral research or research employing survey, interview, oral history, focus group, program evaluation, human factors evaluation, or quality assurance methodologies
- □ Category 8: Continuing review of previously approved full committee research that is essentially complete
- ■ Category 9: Continuing review of research NOT involving an IND or IDE where the IRB has determined that the research poses no greater than minimal risk

6.4	* This study involves:

- ● Subject contact (including phone, email or web contact)
- ○ No subject contact (limited to medical records review, biological specimen analysis, and/or data analysis)

7.0 Funding

7.1 Identify all sponsors and provide the funding details:

External Sponsor:

View Details	Sponsor Name	Sponsor Type	Awardee Institution	Contract Type:	UCSF RAS "P number" or eProposal number	UCSF RAS System Award Number ("A" + 6 digits)
No Sponsor has been added to this Study						

Gift, Program, or Internal Funding (check all that apply):

- ■ Funded by gift (specify source below)
- □ Funded by UCSF or UC-wide program (specify source below)
- □ Specific departmental funding (specify source below, if applicable)
- □ Unfunded (miscellaneous departmental funding)
- □ Unfunded student project

List the gift, program, or departmental funding source:

Private donation to the Osher Center for Integrative Medicine

7.2 If you tried to add a sponsor in the question above and it was not in the list, follow these steps:

- **If funding has already been awarded or the contract is being processed by the Contracts and Grants or Industry Contracts unit, your sponsor is already in the system and the project has a UCSF RAS System Proposal or Award number. Check with your department's Research Services Analyst (RSA) to ask how the sponsor is listed in the UC sponsor list and what the Proposal or Award number is.**
- **If you need additional assistance, contact the Contracts and Grants Award Team at CGAwardTeam@ucsf.edu and list the sponsor in the box below.**

○ Sponsor not in list

Only if your sponsor is not yet in the list, type the sponsor's name:

If the funding is administered by the UCSF Office of Sponsored Research, your study will not receive CHR approval until the sponsor and funding

details have been added to your application.

7.3 * **This study is supported in whole or in part by Federal funding:**

○ Yes ● No

If **yes**, indicate which portion of your grant you will be attaching:

- □ The Research Plan, including the Human Subjects Section of your NIH grant or subcontract
- □ For other federal proposals (contracts or grants), the section of the proposal describing human subjects work
- □ The section of your progress report if it provides the most current information about your human subjects work
- □ The grant is not attached. The study is funded by an award that does not describe specific plans for human subjects, such as career development awards (K awards), cooperative agreements, program projects, and training grants (T32 awards)

8.0 Statement of Financial Interest

8.1 * **The Principal Investigator and/or one or more of the key study personnel has financial interests related to this study:**

○ Yes ● No

If **Yes**, attach the Disclosure of Investigators' Financial Interests Supplement to this application.

9.0 Sites

9.1 Institutions (check all that apply):

- ■ UCSF
- □ Mt. Zion
- □ San Francisco General Hospital (SFGH)
- □ SF VA Medical Center (SF VAMC)
- □ Helen Diller Family Comprehensive Cancer Center
- □ Blood Centers of the Pacific (BCP)
- □ Blood Systems Research Institute (BSRI)
- □ Fresno (Community Medical Center)
- □ Gallo
- □ Gladstone
- ■ Institute on Aging (IOA)
- □ Jewish Home
- □ SF Dept of Public Health (DPH)

9.2 Check all the other types of sites not affiliated with UCSF with which you are cooperating or collaborating on this project:

- □ Other UC Campus

□ Other institution

□ Other community-based site

□ Foreign Country

List the foreign country/ies:

9.3 * **This is a multicenter study:**

○ Yes ● No

9.4 **Check any research programs this study is associated with:**

□ Cancer Center
□ Center for AIDS Prevention Sciences (CAPS)
□ Global Health Sciences
□ Immune Tolerance Network (ITN)
■ Osher Center
□ Positive Health Program

10.0 Study Design

10.1 * **Study design:**

Development (phase 1) and feasibility/pilot testing (phase 2) of an integrative exercise intervention to enable individuals with mild-to-moderate dementia to maintain function and independence.

10.2 **Check all that apply:**

■ Phase I
□ Phase II
□ Phase III
□ Phase IV

11.0 Scientific Considerations

11.1 **Hypothesis:**

This study has a hypothesis:
○ Yes ● No

If yes, state the hypothesis or hypotheses:

11.2 * **List the specific aims:**

1. To develop and refine PLIÉ (Preventing Loss of Independence through Exercise) in 2 to 4 individuals with mild-to-moderate dementia. PLIÉ is a novel, integrative exercise program designed to help individuals with mild-to-moderate dementia maintain independence by integrating elements of traditional strength-building exercises with

Tai Chi, Feldenkrais and yoga (Phase 1).

2. To perform a 36-week pilot test of PLIÉ in approximately 16 individuals with mild-to-moderate dementia in order to examine issues related to compliance and safety and to estimate effect sizes for a larger study (Phase 2).

11.3 Statistical analysis:

Because this is a pilot study, statistics will primarily be descriptive and will include calculation of means, standard deviations and percentages for demographic characteristics and baseline levels of cognitive and physical function. We also will calculate change in key measures (paired t-test, McNemar's test) to enable calculation of effect sizes and sample size estimates for future projects.

11.4 * This is an investigator-initiated study:

● Yes ○ No

11.5 This study has received scientific or scholarly review from (check all that apply):

- ☐ Cancer Center Protocol Review Committee (PRC) (Full approval or contingent PRC approval is required prior to final CHR approval for cancer-related protocols.)
- ☐ CTSI Clinical Research Center (CRC) advisory committee
- ☐ Departmental scientific review
- ☐ Other:

Specify **Other**:

If applicable, attach the Departmental Scientific Review Form at the end of the application.

12.0 Background

12.1 Background:

The dementia epidemic is one of the most important public health issues that our nation will face in the coming years. The number of people with Alzheimer’s disease and other dementias in the United States is currently more than 5 million, and a new person develops the disease every 72 seconds (1). By 2050, it is anticipated that 10 million baby boomers will have developed dementia, resulting in a total of 11 to 16 million cases. The costs of this epidemic—both economically and in terms of the suffering of affected individuals and their loved ones—will make dementia one of the top health issues facing the nation.

The progressive loss of independence is one of the most difficult aspects of Alzheimer’s disease for both affected individuals and caregivers. We have previously found that dependence in one or more basic daily activities is a key predictor of placement in a long-term care facility (2). Current drug treatments are associated with small improvements in cognitive function but have minimal impact on important outcomes such as independence (3).

A handful of small studies have found that exercise interventions improve physical and cognitive function in healthy elders (4) and individuals with mild cognitive impairment (5). However, surprisingly few studies have examined the effects of exercise in individuals with mild to moderate-stage Alzheimer’s disease (6), particularly as a

method for maintaining independence. Exercise programs based on Eastern traditions, such as Yoga and Tai Chi, have shown encouraging results for physical and emotional functioning in the elderly, and improvements in older patients with various chronic conditions, including cardiovascular disease (7). They have rarely been studied in patients with dementia, however.

The goal of our study is to develop and pilot-test an integrative exercise program specifically designed to help older adults with mild-to-moderate dementia maintain independence and function. This program will include traditional strength-building exercises targeting the muscles required for basic daily activities as well as elements of Tai Chi, Feldenkrais and yoga.

12.2 Preliminary studies:

Prior Experience of the Research Team. The research team has previously performed a wide range of RCTs of non-pharmacological interventions in a variety of study populations and settings including in individuals with cognitive deficits.
Dr. Deborah Barnes, Associate Professor of Psychiatry (effective 7/1/2011) at UCSF, will be PI. Dr. Barnes is an epidemiologist with extensive training in RCT methodology. She has recently completed an RCT of computer-based mental activity in individuals with mild cognitive impairment (MCI) as well as the Mental Activity and eXercise (MAX) Trial—an Alzheimer's Association-funded study of the effects of physical and mental activity on cognitive function in sedentary elders with cognitive complaints.
Dr. Kristine Yaffe, Professor of Psychiatry, Neurology and Epidemiology/Biostatistics at UCSF, will serve as a Co-Investigator. Dr. Yaffe is an internationally renowned expert in cognitive impairment and dementia. She has been PI of several RCTs of pharmacological interventions to improve cognitive function and reduce cognitive decline in older adults and has also participated in several RCTs of non-pharmacologic interventions.
Dr. Margaret Chesney, Professor of Medicine and Director of the Osher Center for Integrative Medicine at UCSF, will serve as Co-Investigator. Dr. Chesney was Deputy Director of the National Center of Complimentary and Alternative Medicine at the National Institutes of Health for five years (2003-2008) and has extensive knowledge of alternative approaches to health and well-being.
Dr. Wolf Mehling, Associate Professor of Family and Community Medicine at UCSF, will serve as Co-Investigator. Dr. Mehling has clinical training and experience with a variety of integrative medicine approaches including Feldenkrais, Tai Chi and yoga and has completed several RCTs in individuals with cancer and low back pain.

12.3 References:

1. 2010 Alzheimer's Disease Facts & Figures. 2010. (Accessed January 10, 2011, at http://www.alz.org/documents_custom/report_alzfactsfigures2010.pdf.)

2. Yaffe K, Fox P, Newcomer R, et al. Patient and caregiver characteristics and nursing home placement in patients with dementia. Jama 2002;287:2090-7.

3. O'Brien JT, Burns A. Clinical practice with anti-dementia drugs: a revised (second) consensus statement from the British Association for Psychopharmacology. J Psychopharmacol 2010;[Epub ahead of print].

4. Heyn P, Abreu BC, Ottenbacher KJ. The effects of exercise training on elderly persons with cognitive impairment and dementia: a meta-analysis. Arch Phys Med Rehabil 2004;85:1694-704.

5. Lautenschlager NT, Cox KL, Flicker L, et al. Effect of physical activity on cognitive function in older adults at risk for Alzheimer disease: a randomized trial. Jama

2008;300:1027-37.

6. Forbes D, Forbes S, Morgan DG, Markle-Reid M, Wood J, Culum I. Physical activity programs for persons with dementia. Cochrane database of systematic reviews (Online) 2008:CD006489.

7. Rogers CE, Larkey LK, Keller C. A review of clinical trials of tai chi and qigong in older adults. West J Nurs Res 2009;31:245-79.

If you have a separate bibliography, attach it to the submission with your other study documents.

13.0 Sample Size and Eligibility

13.1 Number of subjects that will be enrolled at UCSF and affiliated institutions (locally):

20

13.2 Total number of subjects that will be enrolled at all sites (for study overall):

20

13.3 Estimated number of people that you will need to consent and screen here (but not necessarily enroll) to get the needed subjects:

24

13.4 Sample size calculation:

The primary goal of this pilot study is to assess the safety and feasibility of PLIÉ. As described in more detail below, we will begin by beta-testing the program in 2-4 individuals (Phase 1). We then will pilot-test the program in 16 subjects who will be randomized to either Group 1 (intervention) or Group 2 (wait-list control) (Phase 2). Sample sizes were based on prior experiences in the field suggesting a maximum class size of 8 subjects with mild-to-moderate dementia and 2 instructors.

13.5 * Eligible age range(s):

- ☐ 0-6 years
- ☐ 7-12 years
- ☐ 13-17 years
- ■ 18+ years

13.6 Inclusion criteria:

Primary participant: Age >=55 years, mild-to-moderate dementia (defined as physician diagnosis of dementia and Mini-Mental State Examination (MMSE) score of 10-26), English language fluency, ability to walk independently without a walker or cane, physician approval and caregiver consent to participate.

Caregivers: Provide care to primary participant; can provide information about the

primary participant's physical functioning, behaviors, falls, quality of life and own level of stress.

13.7 Exclusion criteria:

Primary participant: Other neurologic disease (e.g., stroke, Parkinson’s disease, ALS), major current psychiatric illness (e.g., schizophrenia, bipolar disorder), life expectancy < 1 year (e.g., metastatic cancer), or inability to assent to study procedures.

Caregiver: Any major neurologic disease (e.g., dementia, stroke, Parkinson’s disease, ALS), major current psychiatric illness (e.g., schizophrenia, bipolar disorder), life expectancy < 1 year (e.g., metastatic cancer), evidence of cognitive impairment or inability to consent to study procedures.

13.8 There are inclusion or exclusion criteria based on gender, race or ethnicity:

○ Yes ● No

If **yes**, please explain the nature and rationale for the restrictions:

14.0 Drugs and Devices

14.1 * Drugs or biologics will be studied under this application:

○ Yes ● No

14.2 * Investigational medical devices or in vitro diagnostics will be used OR approved medical devices or in vitro diagnostics will be studied under this application:

○ Yes ● No

14.3 * A Non-Significant Risk (NSR) determination is being requested for an investigational device:

○ Yes ● No

14.4 Verification of IND/IDE numbers: If the sponsor’s protocol does not list the IND/IDE number, you must submit documentation from the sponsor or FDA identifying the IND/IDE number for this study. Attach this documentation in the Other Study Documents section of the Initial Review Submission Packet.

15.0 Other Approvals and Registrations

15.1 * This is a clinical trial:

● Yes ○ No

Clinical Trial Registration
"NCT" number for this trial:

Study will be registered and NCT number entered following CHR approval.

15.2 * Data from this study will be submitted to NIH for Genome-Wide Association Studies

(GWAS):

○ Yes ● No

15.3 * This study involves vaccines produced using recombinant DNA technologies:

○ Yes ● No

15.4 * This study involves human gene transfer (NOTE: Requires NIH Recombinant DNA Advisory Committee (RAC) review prior to CHR approval):

○ Yes ● No

15.5 * The study protocol requires radiological procedures (e.g. CT scans, x-rays) or exposes subjects to radiation:

○ Yes ● No

15.6 This study involves other regulated materials and requires approval and/or authorization from the following regulatory committees:

□ Institutional Biological Safety Committee (IBC)

Specify BUA #:

□ Institutional Animal Care and Use Committee (IACUC)

Specify IACUC #:

□ Radiation Safety Committee

Specify RUA #:

□ Radioactive Drug Research Committee (RDRC)

Specify RDRC #:

□ Controlled Substances

16.0 Procedures

16.1 * List all study procedures, test and treatments required for this study:

1. Recruitment. Primary participants and caregivers will be recruited as dyads from the Institute on Aging (IOA) Swindells Center for Adult Day Services, a social day program for individuals with mild-to-moderate memory loss. The program runs Monday - Friday from 9:30-3:30 and includes meals and transportation to and from the participant's home. There are currently approximately 45 clients that attend 2-5 days/week with 30 attending on a given day. The IOA Director, Dr. David Werdeger, and the Swindells Director, Genya Boyko, have both approved use of their clients and facilities for this pilot study.

IOA staff will determine initial eligibility (e.g., primary participant age, diagnosis,

English language fluency, ability to walk) and will contact caregivers of potential participants to let them know about the study. A letter will then be sent to caregivers of potential participants describing the study and providing an opportunity to learn more by returning a form, calling the research study or talking with IOA staff. Those caregivers who express interest will be contacted by research staff by phone to further assess interest/eligibility and to schedule a consent visit.

2. Informed consent. As described in more detail below, primary participants and caregivers will participate in the consent process together. The consent forms will be reviewed and capacity to consent will be assessed in primary participants based on responses to questions to determine level of understanding of study procedures, risks, benefits and rights. Participants who do not demonstrate capacity to consent will be asked to assent to study procedures and caregivers will be asked to consent on their behalf. After the consent process has been completed, the Mini-Mental State Exam will be administered to primary participants to assess level of cognitive impairment. Consent procedures will take place at IOA unless otherwise requested by caregivers.

3a. **Phase 1: Beta-test**. Two to 4 participant/caregiver dyads will participate in refining the exercise protocol. We have developed PLIÉ in consultation with experts worldwide who have experience performing different types of exercises in individuals with mild-to-moderate dementia, including traditional strength-building exercises, yoga, Tai Chi and Feldenkrais. Specifically, we have identified exercises from each of these traditions that engage the muscles most needed to maintain independence--including lower body strength (to help with getting out of bed/chair), balance (to minimize risk of falls), upper body strength (to help with lifting), fine motor exercises (to help with activities such as eating and brushing teeth), and pelvic floor exercises (to help with continence)--and we have combined them into a unique integrative exercise program. These exercises are designed to be purposeful (i.e., to achieve a goal) and to build procedural ('muscle') memory. In addition, breathing and guided meditation exercises are included to promote relaxation and well-being.

Because we are combining these exercises in a new way, we feel that it is important for us to beta-test and refine the program in a small group of subjects prior to pilot-testing it in a larger group. The primary goal of the beta-testing phase will be to identify a core group of exercises that target the key muscles and movements required to maintain functional independence that are safe, fun and meaningful to the participants as well as variations of these exercises to accomodate differences in baseline functional ability of participants. Classes will occur 2-3 days/week for 30-45 minutes for 8 weeks. Caregivers will be invited to attend 1 class per month, but this will be optional.

3b. Phase 2: Pilot-test. Participant/caregiver dyads who do not participate in beta-testing will be invited to participate in the pilot test. These subjects will be randomly assigned to either Group 1 (intervention), which will participate first, or Group 2 (waitlist control group). Group 1 will participate in the PLIÉ program 2-3 days/week for 30-45 minutes for 18 weeks while Group 2 engages in their usual activities at the day care center, which includes a brief daily chair-based exercise session. After Group 1 has completed their training, Group 2 will participate in the PLIÉ program 2-3 days/week for 30-45 minutes for 18 weeks. Caregivers will be invited to attend 1 class per month, but this will be optional.

4. Pre- and post-intervention assessment. We will perform a variety of standard measures in all participants and caregivers. In the beta-testing group, assessments will occur at baseline and after the 8-week training period and will primarily be utilized to assess feasibility and logistics of data collection procedures. In the pilot-testing group, assessments will be performed at baseline, 18 weeks and 36 weeks in all participants. Separate appointments will be made for primary participants and caregivers to maximize the validity of data collected (e.g., to ensure that

caregivers do not 'help' primary participants with answers to questions and feel comfortable providing information about issues such as difficult behaviors or levels of stress). Assessments will take place at IOA unless otherwise requested by caregivers.

***4a. Primary participants*.**

Cognitive function will be assessed with the Alzheimer’s Disease Assessment Scale – Cognitive Subscale (ADAS-cog) (Rosen 1984), which is the most commonly used primary outcome measure in AD treatment trials. It is an 80-point scale that includes direct assessment of learning (word list), naming (objects), following commands, constructional Praxis (figure copying), ideational Praxis (mailing a letter), orientation (person, time, place), recognition memory and remembering test instructions.

Physical performance will be assessed with the Short Physical Performance Battery (SPPB), which was developed by the National Institute on Aging to provide an objective tool for evaluating lower extremity functioning in older adults. The test includes repeated chair stands, tandem balance testing and 8’ walking speed (Guralnik 1994).

Fall-related self-efficacy will be assessed with the Falls Efficacy Scale (FES), which is a 10-item scale that has been validated in individuals with cognitive impairment (Hauer 2010).

Quality of life will be assessed with the Quality of Life Scale in Alzheimer’s Disease (QOL-AD), which is a brief, 13-item measure that obtains input from both the individual and the caregiver (Logsdon 1999). Scores may range from 13-52 points.

Functional health and well-being will be assessed with the SF-36v2, which assesses function in eight domains and provides physical and mental component summary scores that may range from 0 to 70 (Stewart 1989). The SF-36v2 is typically administered directly to participants. However, we also will ask caregivers to answer questions on behalf of participants and will compare their answers to assess congruence. Data collected will help to determine if this measure is suitable for a larger trial.

***4b. Caregivers*.**

Participant's physical function will be assessed with the Alzheimer’s Disease Cooperative Study - Activities of Daily Living (ADCS-ADL) scale (Galasko 1997). The ADCS-ADL is a 78-point scale that assesses functional ability in 23 daily activities based on informant report. It is the standard measure for assessment of functional status in AD treatment trials.

Participant's dementia-related behaviors will be assessed with the Neuropsychiatric Inventory (NPI), which is a 144-point informant-based questionnaire that assesses 12 behavioral domains common in dementia including frequency, severity and impact on caregiver distress (Cummings 1997).

Participant's falls will be assessed based on caregiver report of number of falls in the previous 8 weeks (beta-testing group) or 18 weeks (pilot-testing groups).

Participant's quality of life will be assessed with the Quality of Life Scale in Alzheimer’s Disease (QOL-AD), which is a brief, 13-item measure that obtains input from both the individual and the caregiver (Logsdon 1999). Scores may range from 13-52 points.

Participant and caregiver functional health and well-being will be assessed

with the SF-36v2, which assesses function in eight domains and provides physical and mental component summary scores that may range from 0 to 70 (Stewart 1989). The SF-36v2 is typically administered directly to participants. However, we also will ask caregivers to answer questions on behalf of participants and will compare their answers to assess congruence.

Caregiver burden will be assessed with the Caregiver Burden Inventory (CBI), which is a 96-point scale that includes 24 items and 5 domains (Novak 1989).

5. Goal-setting.

Prior to beginning PLIÉ, participants and caregivers will talk with the exercise instructor to discuss which daily activities are most meaningful to them and to determine whether there are any specific goals related to physical function or daily activities that they would like to achieve. The exercise instructor will use this information to target exercises toward these goals during training if possible. Discussions will be in person with the participant and may be in person or by phone with the caregiver.

6. Compliance and adverse events. Compliance will be assessed based on class attendance. Bi-weekly check-in calls will be performed with caregivers to assess for adverse events and to address issues related to non-compliance.

If you have a procedure table, attach it to the submission with your other study documents.

**16.2**	**Interviews, questionnaires, and/or surveys will be administered or focus groups will be conducted:**

● Yes ○ No

List any standard instruments used for this study:

- Mini-Mental State Examination (MMSE) (Folstein 1975)
- Alzheimer’s Disease Cooperative Study - Activities of Daily Living (ADCS-ADL) scale (Galasko 1997)
- Alzheimer’s Disease Assessment Scale – Cognitive Subscale (ADAS-cog) (Rosen 1984)
- Physical Performance Battery (SPPB) (Guralnick 1994)
- Neuropsychiatric Inventory (NPI) (Cummings 1997)
- Falls Efficacy Scale (FES) (Hauer 2010)
- Quality of Life Scale in Alzheimer’s Disease (QOL-AD) (Logsdon 1999)
- SF-36v2 (Stewart 1989)
- Caregiver Burden Inventory (CBI) (Novak 1989)

Attach any non-standard instruments at the end of the application.

**16.3**	**Conduct of study procedures or tests off-site by non-UCSF personnel:**

● Yes ○ No

If yes, explain:

Study procedures will be performed at the Institute on Aging's Irene Swindells Center for Adult Day Services by UCSF personnel.

**16.4**	**Sharing of experimental research test results with subjects or their care providers:**

○ Yes ● No

If yes, explain:

16.5 * **Specimen collection for future research and/or specimen repository/bank administration:**

○ Yes ● No

16.6 **Time commitment (per visit and in total):**

Primary participant

- Consent/eligibility visit (30-45 minutes)
- Baseline assessment (60-90 minutes)
- Intervention (30-45 minutes, 2-3 days/week for either 8 weeks (Phase 1 beta-testing) or 18 weeks (Phase 2 pilot-testing))
- Post-intervention assessments at 18 and 36 weeks (60-90 minutes each)
- Total: 12-20 hours over 10 weeks for the beta-testing group; 20-45 hours over 40 weeks for pilot-study group

Caregiver

- Consent/eligibility visit (30-45 minutes)
- Baseline assessment (60-90 minutes)
- Biweekly telephone check-ins (5-10 minutes each)
- Post-intervention assessments at 18 and 36 weeks (60-90 minutes each)
- Total: 4-6 hours over 10 weeks for the beta-testing group; 5-7 hours over 40 weeks for pilot-study group

16.7 **Locations:**

All study procedures will take place at the Institute on Aging's Irene Swindells Center for Adults Day Services in San Francisco.

16.8 **Describe the resources in place to conduct this study in a way that assures protection of the rights and welfare of participants:**

The safety and welfare of study participants will be ensured by performing all study procedures at the IOA, where they are already attending an adult day care program. Study personnel who assess for eligibility, obtain consent and administer study questionnaires will be extensively trained in how to work with individuals with mild-to-moderate dementia and caregivers in a sensitive and respectful manner. Class size will be limited to 2-4 participants and 1 instructor during beta-testing and 8 participants with 2 instructors during pilot-testing to ensure the safety of study participants. Exercise instructors will be certified in at least one type of exercise training and will be extensively trained in how to safely work with individuals with mild-to-moderate dementia. Data will be maintained on secure servers or in locked file cabinets.

17.0 Alternatives

17.1 **Study drug or treatment is available off-study:**

○ Yes
○ No
● Not applicable

17.2 * Is there a standard of care (SOC) or usual care that would be offered to prospective subjects at UCSF (or the study site) if they did not participate:

● Yes ○ No

If yes, describe the SOC or usual care that patients would receive if they choose not to participate:

The adult day health program at IOA currently includes approximately 20 minutes of chair-based exercises. These classes will be available to individuals who choose not to participate in our study or are assigned to the wait-list control group.

17.3 Describe other alternatives to study participation that are available to prospective subjects:

Subjects could ask their physicians to suggest other exercise programs or could engage in similar exercises on their own at home. They also could choose not to participate.

18.0 Risks and Benefits

18.1 * Risks and discomforts:

As with any program involving new exercises, primary participants may experience injuries including muscle strain or soreness, joint pain or fall-related injuries. In addition, primary participants or caregivers may find the baseline or follow-up assessments to be stressful. Loss to privacy may occur if information is released outside the study team.

18.2 Steps taken to minimize risks to subjects:

We have consulted with a wide range of experts who have experience leading exercise classes for individuals with mild-to-moderate dementia to ensure that the PLIE exercise program is safe and likely to be effective. These include local, national and international experts in traditional exercise programs as well as Tai Chi, Feldenkrais, and yoga. We will minimize stress during baseline and post-intervention assessments by training research staff to recognize and appropriately address signs of discomfort or stress when indicated (e.g., taking breaks, rescheduling appointment, skipping sections that cause undue discomfort). We will minimize risk of loss of privacy by training all research staff to maintain data in a secure manner and not to discuss study participants outside the research team.

18.3 Benefits to subjects:

● Yes ○ No

If yes, describe:

Some primary participants may experience improvements in physical, emotional or cognitive function or sleep, but this is not guaranteed.

18.4 Benefits to society:

We anticipate that findings from this pilot study will be used to support a larger study that will determine the efficacy of PLIE for helping individuals with mild-to-moderate dementia maintain function and independence. If this program is successful, it could substantially improve quality of life for individuals with dementia and their caregivers.

18.5 Explain why the risks to subjects are reasonable:

The risks to subjects are comparable to daily life experiences related to engaging in physical activity or in being assessed in a clinical setting.

19.0 Confidentiality and Privacy

19.1 Plans for maintaining privacy in the research setting:

Data will be entered into the MyResearch portal, which provides a secure web-based data management and storage system for UCSF investigators. Paper copies of data collection forms will be stored in locked files cabinets. Subjects will be identified by a unique participant identification number. Personal identifying information will be stored separately and securely.

19.2 Possible consequences to subjects resulting from a loss of privacy:

Loss of privacy could potentially result in stigma related to dementia diagnoses or test scores.

19.3 Study data are:

- ☐ Derived from the Integrated Data Repository (IDR) or The Health Record Data Service (THREDS) at SFGH
- ☐ Derived from a medical record (identify source below)
- ☐ Added to the hospital or clinical medical record
- ☐ Created or collected as part of health care
- ☐ Used to make health care decisions
- ■ Obtained from the subject, including interviews, questionnaires
- ☐ Obtained from a foreign country or countries only
- ☐ Obtained from records open to the public
- ☐ Obtained from existing research records
- ☐ None of the above

If **derived from a medical record**, identify source:

19.4 Identifiers may be included in research records:

● Yes ○ No

If **yes**, check all the identifiers that may be included:

- ■ Names
- ■ Dates
- ■ Postal addresses
- ■ Phone numbers
- ☐ Fax numbers

■ Email addresses
□ Social Security Numbers*
■ Medical record numbers
■ Health plan numbers
□ Account numbers
□ License or certificate numbers
□ Vehicle ID numbers
□ Device identifiers or serial numbers
□ Web URLs
□ IP address numbers
□ Biometric identifiers
□ Facial photos or other identifiable images
□ Any other unique identifier

* Required for studies conducted at the VAMC

19.5 Identifiable information might be disclosed as part of study activities:

● Yes ○ No

If **yes**, indicate to whom identifiable information may be disclosed:

□ The subject's medical record
□ The study sponsor
□ Collaborators
□ The US Food & Drug Administration (FDA)
■ Others (specify below)
□ A Foreign Country or Countries (specify below)

If **Others**, specify:

Medical personnel if there is a study-related injury. Study auditors.

19.6 Indicate how data are kept secure and protected from improper use and disclosure (check all that apply):

NOTE: Whenever possible, do not store subject identifiers on laptops, PDAs, or other portable devices. If you collect subject identifiers on portable devices, you MUST encrypt the devices.

■ Data are stored securely in My Research
□ Data are coded; data key is destroyed at end of study
■ Data are coded; data key is kept separately and securely
■ Data are kept in a locked file cabinet
■ Data are kept in a locked office or suite
■ Electronic data are protected with a password
■ Data are stored on a secure network
■ Data are collected/stored using REDCap or REDCap Survey

19.7 Additional measures to assure confidentiality and protect identifiers from improper use and disclosure, if any:

Research staff are HIPAA-trained and will be instructed not to discuss or disclose information about study participants outside the research team.

19.8 This study may collect information that State or Federal law requires to be reported to other officials or ethically requires action:

● Yes ○ No

Explain:

Interviews with subjects or caregivers could potentially reveal information related to abuse or thoughts of suicide or homicide.

19.9 This study will be issued a Certificate of Confidentiality:

○ Yes ● No

20.0 Subjects

20.1 Check all types of subjects that may be enrolled:

□ Inpatients
□ Outpatients
■ Healthy volunteers
□ Staff of UCSF or affiliated institutions

20.2 Additional vulnerable populations:

□ Children
□ Subjects unable to consent for themselves
□ Subjects unable to consent for themselves (emergency setting)
■ Subjects with diminished capacity to consent
□ Subjects unable to read, speak or understand English
□ Pregnant women
□ Fetuses
□ Neonates
□ Prisoners
□ Economically or educationally disadvantaged persons
□ Investigators' staff
□ Students

Explain why it is appropriate to include the types of subjects checked above in this particular study:

The goal of the study is to determine whether the PLIE exercise program enables individuals with mild-to-moderate dementia to maintain functional status and independence. Therefore, although study participants will have a diminished capacity to provide consent, findings from the study could potentially improve quality of life for some study participants and provide important data for others with mild-to-moderate dementia.

Describe the additional safeguards that have been included in the study to protect the rights and welfare of these subjects and minimize coercion or undue influence:

Informed consent will be obtained from study participants and their caregivers

together. Capacity to consent will be determined using a standard protocol that is based on the protocol used at the UCSF Memory and Aging Center. Subjects who are able to consent for themselves will do so. Subjects who do not demonstrate capacity to consent will be asked for assent and caregivers will provide consent on their behalf.

21.0 Recruitment

21.1 * Methods (check all that apply):

- □ Study investigators (and/or affiliated nurses or staff) recruit their own patients directly in person or by phone.
- □ Study investigators recruit their own patients by letter. Attach the letter for review.
- □ Study investigators send a "Dear Doctor" letter to colleagues asking for referrals of eligible patients. If interested, the patient will contact the PI or the PI may directly recruit the patients (with documented permission from the patient). Investigators may give the referring physicians a study information sheet for the patients.
- □ Study investigators provide their colleagues with a "Dear Patient" letter describing the study. This letter can be signed by the treating physicians and would inform the patients how to contact the study investigators. The study investigators may not have access to patient names and addresses for mailing
- □ Advertisements, notices, and/or media used to recruit subjects. Interested subjects initiate contact with study investigators. Attach ads, notices, or media text for review. In section below, please explain where ads will be posted.
- □ Study investigators identify prospective subjects through chart review. (Study investigators request a Waiver of Authorization for recruitment purposes.)
- □ Large-scale epidemiological studies and/or population-based studies: Prospective subjects are identified through a registry or medical records and contacted by someone other than their personal physician. (Study investigators request a Waiver of Authorization for recruitment purposes.)
- □ Direct contact of potential subjects who have previously given consent to be contacted for participation in research. Clinic or program develops a CHR-approved recruitment protocol that asks patients if they agree to be contacted for research (a recruitment database) or consent for future contact was documented using the consent form for another CHR-approved study.
- □ Study investigators list the study on the School of Medicine list of UCSF Clinical Trials website or a similarly managed site. Interested subjects initiate contact with investigators.
- □ Study investigators recruit potential subjects who are unknown to them through methods such as snowball sampling, direct approach, use of social networks, and random digit dialing.
- ■ Other

If **Other**, explain:

As described in the Procedures section, IOA staff will assess initial eligibility based on factors such as the primary participant's age, diagnosis and ability to walk independently. Caregivers of potentially eligible subjects will be contacted by IOA staff either in person or by telephone to make them aware of the study and to let them know they will be receiving a letter in the mail. Study investigators will then mail a letter to caregivers describing the study and providing an opportunity to participate by returning a form, calling the research office or telling IOA staff.

21.2 * How, when, and by whom eligibility will be determined:

Initial eligibility (age, dementia diagnosis, English language fluency, ability to walk independently) will be determined by IOA staff. Caregivers who express interest will be

contacted by telephone by research staff to assess initial eligibility criteria. Those meet initial criteria will be scheduled for an appointment to obtain written consent and to assess other eligiblity criteria (mild-to-moderate stage disease based on Mini-Mental State Exam score).

21.3 * **How, when, where and by whom potential subjects will be approached:**

Please see above.

21.4 * **Protected health information (PHI) will be accessed prior to obtaining consent:**

● Yes ○ No

22.0 Waiver of Consent/Authorization for Recruitment Purposes

(Note: This section partially replaces the old "Request for Waiver of Consent/Authorization for Minimal Risk Research or for Screening for Recruitment" supplement form. Please do not attach the old form to this application.)

This section is now required when study investigators (and/or affiliated nurses or staff) recruit their own patients directly.

22.1 * **Study personnel need to access protected health information (PHI) during the recruitment process and it is not practicable to obtain informed consent until potential subjects have been identified:**

● Yes

If **no**, a waiver of consent/authorization is NOT needed.

22.2 * **A waiver for screening of health records to identify potential subjects poses no more than minimal risk to privacy for participants:**

● Yes

If **no**, a waiver of authorization can NOT be granted.

22.3 * **Screening health records prior to obtaining consent will not adversely affect subjects' rights and welfare:**

● Yes

If **no**, a waiver of authorization can NOT be granted.

22.4 * **Check all the identifiers that will be collected prior to obtaining informed consent:**

- Names
- Dates
- Postal addresses

- ■ Phone numbers
- □ Fax numbers
- □ Email addresses
- □ Social Security Numbers*
- □ Medical record numbers
- □ Health plan numbers
- □ Account numbers
- □ License or certificate numbers
- □ Vehicle ID numbers
- □ Device identifiers or serial numbers
- □ Web URLs
- □ IP address numbers
- □ Biometric identifiers
- □ Facial photos or other identifiable images
- □ Any other unique identifier
- □ None

Note: HIPAA requires that you collect the minimum necessary.

22.5 * Describe any health information that will be collected prior to obtaining informed consent:

During the telephone screening process, we will collect data to assess initial eligibility criteria including diagnoses of exclusionary medical conditions or contraindications to exercise.

Note: HIPAA requires that you collect the minimum necessary.

22.6 * Describe your plan to destroy the identifiers at the earliest opportunity consistent with the research or provide a health or research justification for retaining the identifiers, or indicate and explain that retention is required by law:

PHI for individuals who decline to participate will be retained to ensure that these individuals are not contacted inadvertently for future studies.

23.0 Informed Consent

23.1 * Methods (check all that apply):

- ■ Signed consent will be obtained from subjects and/or parents (if subjects are minors)
- □ Verbal consent will be obtained from subjects using an information sheet or script
- □ Electronic consent will be obtained from subjects via the web or email
- □ Implied consent will be obtained via mail, the web or email
- ■ Signed consent will be obtained from surrogates
- □ Emergency waiver of consent is being requested for subjects unable to provide consent
- □ Informed consent will not be obtained

23.2 * Process for obtaining informed consent:

The participant and caregiver will sit together with research staff during the consent process. Research staff will review the key points of the consent documents and will ask if the participant or caregiver have any questions. Research staff will then ask questions of the participant to assess capacity to consent. The assessment form is included for review. If the participant demonstrates capacity to consent, they will sign the consent form; if not, they will be informed that the caregiver is being asked to sign on their behalf, and the participant will be asked to assent to study procedures. Because caregivers also will be actively participating in the study (by providing information about the participant behaviors, stress, etc.), they will sign a separate consent form. Finally, research staff will sign both forms and will indicate that an assent discussion was completed if appropriate.

23.3 * **How investigators will make sure subjects understand the information provided to them:**

Research staff will ask participants a series of yes/no questions to assess whether consent information was understood. The wording of the questions will be varied to ensure that participants cannot simply answer yes or no to all questions. Information not understood will be discussed again and understanding will be reassessed. If participants are unable to answer any questions after two discussions, they will be considered unable to provide consent and will be asked to assent to study procedures by agreeing to have their caregiver provide consent on their behalf. Participants who are unable to provide either consent or assent will be considered ineligible for the study.

24.0 Surrogate Consent

(Note: This section partially replaces the old "Surrogate Consent" supplement form. Please do not attach the old form to this application.)

24.1 Subjects are inpatients on a psychiatric ward or mental health facility, or on psychiatric hold:

- No

If **yes**, use of surrogate consent for research is NOT allowed in California.

24.2 This study is related to the cognitive impairment, lack of capacity, or serious or life-threatening diseases and conditions of the research subjects:

- Yes

If **no**, use of surrogate consent for research is NOT allowed in California.

24.3 Explain why use of surrogates is necessary for completion of this study:

Purpose of the study is to develop an exercise program that will help to delay loss of independence in individuals with mild to moderate dementia. Consequently, due to the nature and purpose of the study, it is likely that some participants may have lost their capacity for decision making. A surrogate may be necessary for multiple reasons: A) to be able to provided consent should it be determined that the study participant does not have the capacity to make decisions and B) to provide reliable information regarding the participant’s current level of functioning including mental and physical functioning, relevant to the study and C) to provide information on caregiver burden resulting from the loss of independence of the participant.

24.4	Plans for assessing the decision-making capacity of prospective subjects:

1. Attempts will be made to obtain informed consent directly from the subject.
2. To ensure the subject has the capacity to consent and is able to directly consent, decision making capacity will be assessed by interviewer who will be thoroughly trained by the PI.
3. Subject's decision making capacity will be assessed by observations made during the consent and information sharing process. Observations made will be used to assess the subject's ability:
    1. to make and state a decision and
    2. to understand the study objectives, in particular the potential participant's ability to express his/her own understanding and rationale for decisions involving:
        - The nature of the research and relevant information including time in the study and procedures.
        - Consequences of not participating in the study
        - Alternatives to participation
4. A Capacity Assessment Record for PLIÉ participants will be used to record the capacity to consent and will be kept with the research chart.
5. Should the investigator determine the subject lacks decision making capacity, the investigator will inform the participant and seek surrogate consent.
6. Surrogate will then be asked to fill out the Self-Certification of Surrogate Decision Maker form. The form will be kept in the participant's research chart.
7. Consent form will be reviewed again with surrogate and participant.
8. Consent will be obtained from surrogate
9. Assent will be obtained from participant
10. Should the participant not assent, the participant will be excluded from the study despite the surrogate's consent.

24.5	Plans for obtaining consent from subjects who regain ability to consent after a surrogate has given initial consent:

Dementia progression is typically assessed in clinical and research settings every 6 to 12 months. Because our study is low-risk and relatively brief (8-36 weeks), we will not reassess capacity to consent in participants. However, a subject's resistance or objection to engaging in study procedures at any point will be taken as evidence of refusal or withdrawal.

24.6	Requirements for any study involving surrogates for obtaining informed consent. Check to acknowledge:

- ■ Research takes place in California. All surrogates will complete the "Self-Certification of Surrogate Decision Makers for Participation in Research" form.
- ■ Conscious subjects will be notified of the decision to contact a surrogate. If subjects object to study participation, they will be excluded even if their surrogate has given consent.
- ■ Surrogates will not receive any financial compensation for providing consent.
- ■ If a higher-ranking surrogate is identified at any time, the investigators will defer to the higher-ranking surrogate's decision regarding the subject's participation in the research.

For research taking place outside of California, explain how investigators will confirm that surrogates are legally authorized representatives:

24.7	VA Studies Only

Provide any additional information to explain comply with the additional VAMC requirements for use of surrogates in research:

25.0 Financial Considerations

25.1 Subjects payment or compensation method (check all that apply):

Payments will be (check all that apply):

■ Subjects will not be paid
□ Cash
□ Check
□ Gift card
□ Other:

Specify **Other**:

25.2 Describe the schedule and amounts of payments, including the total subjects can receive for completing the study. If deviating from recommendations in Subject Payment Guidelines, include specific justification below.

25.3 Costs to Subjects: Will subjects or their insurance be charged for any study procedures?

○ Yes ● No

If **yes,** describe those costs below, and compare subjects' costs to the costs associated with alternative care off-study. Finally, explain why it is appropriate to charge those costs to the subjects.

26.0 CTSI Screening Questions

26.1 * This study will be carried out at one of the UCSF Clinical Research Services (CRS) units or will utilize CRS services:

○ Yes ● No

26.2 This project involves community-based research:

● Yes ○ No

26.3 This project involves practice-based research:

○ Yes ● No
